# ERK‐Mediated Phosphorylation of YAP Defines a Noncanonical FGF Signaling Mechanism in Stem Cells

**DOI:** 10.1002/advs.202511484

**Published:** 2026-04-10

**Authors:** Xiaolei Zhao, Shannon Erhardt, Li Tang, Xiaotong Chen, Stephen M Farmer, Zixiu Cheng, Wen Chen, Ella Ziyuan Lu, Kihan Sung, Chang‐Ru Tsai, Mingjie Zheng, Sheng Zhang, Yang Liu, Jianxin Wang, Min Li, James F Martin, Jun Wang

**Affiliations:** ^1^ Department of Pediatrics McGovern Medical School University of Texas Health Science Center At Houston (UTHealth) Houston Texas USA; ^2^ Department of Diagnostic and Biomedical Sciences School of Dentistry, UTHealth Houston Texas USA; ^3^ MD Anderson Cancer Center UTHealth Graduate School of Biomedical Sciences University of Texas Houston Texas USA; ^4^ (Currently) Department of Genetics Yale School of Medicine New Haven Connecticut USA; ^5^ Hunan Provincial Key Lab on Bioinformatics School of Computer Science and Engineering Central South University Changsha Hunan China; ^6^ Department of Integrative Physiology Baylor College of Medicine One Baylor Plaza Houston Texas USA; ^7^ The Brown Foundation Institute of Molecular Medicine McGovern Medical School At UTHealth Houston Texas USA; ^8^ Department of BioSciences Rice University Houston Texas USA; ^9^ (Currently) Harvard School of Dental Medicine Harvard University Boston Massachusetts USA; ^10^ (Currently) Department of Cardiology The First Affiliated Hospital Zhejiang University School of Medicine Hangzhou China; ^11^ Department of Neurobiology and Anatomy McGovern Medical School At UTHealth Houston Texas USA; ^12^ Department of Integrative Biology and Pharmacology McGovern Medical School At UTHealth Houston Texas USA; ^13^ Cardiomyocyte Renewal Laboratory Texas Heart Institute Baylor College of Medicine Houston Texas USA

**Keywords:** ERK, FGF signaling, neural crest cell, suture mesenchymal cells, YAP

## Abstract

While Fgf and Hippo‐Yap signaling are fundamental for proper development, homeostasis, and disease, their crosstalk remains largely unknown. Here, we identified that Yap and Taz, canonical Hippo effectors, function as noncanonical effectors of Fgf signaling to maintain the proper function of neural crest (NC) lineages. NC cells are a multipotent stem cell population during vertebrate embryogenesis that contribute to numerous structures and diverse cell lineages, including craniofacial and cardiac tissues, neurons, and suture mesenchymal cells (SMCs), a specified cell population required for cranial bone growth and repair. We observed that activation of Fgf signaling in NC cells and NC‐derived SMCs inhibited osteogenesis while simultaneously enhancing stemness and proliferation. Interestingly, these effects were reversed by inhibition of either Yap/Taz or phosphorylated Erk1/2 (pErk1/2). Mechanistically, Fgf signaling promotes the interaction of Yap and pErk1/2, increasing the chromatin occupancy of Yap at genes regulating stemness, proliferation, and osteogenesis. We further show that pERK1/2 phosphorylates YAP at the noncanonical S128 site, enhancing YAP's nuclear localization. This mechanism is conserved across mouse and human cells and is active in Apert syndrome‐associated FGF gain‐of‐function models, revealing a previously unrecognized FGF‐YAP axis in stem cell regulation.

## Graphical abstract

With a powerful combination of in vivo, ex vivo, and in vitro models, the authors highlight a novel FGF‐ERK signaling regulation of YAP at the S128 site in neural crest‐derived stem cells. This study opens exciting new directions in stem cell biology and craniofacial biology, paving the way for potential innovations in the treatment of craniofacial disorders and beyond.

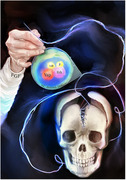

## 1 Introduction

The neural crest (NC), a transient multipotent stem cell population regarded as the “fourth germ layer” during vertebrate embryogenesis, is a powerful model for understanding stem cell plasticity, lineage commitment, and tissue regeneration during vertebrate development [1]. NC cells exhibit a remarkable capacity for multilineage differentiation, such as osteoblasts, chondrocytes, smooth muscle cells, and neurons, generating a wide variety of derivatives for the formation of various tissues/organs, including the majority of the craniofacial skeleton [2, 3]. In calvarial sutures, the NC contributes to suture mesenchymal cells (SMCs) [4] to maintain suture patency and cranial bone growth, repair, and regeneration [5, 6]. Abnormal SMC development during cranial suture formation is a primary cause of craniosynostosis [5, 7, 8, 9], a common birth defect defined by premature cranial suture fusion leading to craniofacial anomalies and neurocognitive impairments [7]. NC‐derived SMCs have recently been indicated as the major contributor to cranial bone repair and regeneration in adult mice, evidenced by efficient NC‐derived frontal bone healing, compared to the mesoderm‐derived parietal bone, upon injury [10]. Furthermore, NC‐derived SMCs showed higher intrinsic proliferation and osteogenic abilities than mesoderm‐derived SMCs in cranial sutures [4, 11]. Despite these promising findings, a significant knowledge gap remains in understanding the molecular regulation of balancing stemness and osteogenesis in NC‐derived SMCs.

Fgf signaling, which is highly conserved between humans and mice, plays essential roles in proliferation, stemness, survival, and differentiation of progenitor or stem cells. For example, in human cells, the FGF ligand FGF2 preserved stem cell pluripotency and self‐renewal capacity [12]. Furthermore, ERK1/2, the canonical downstream effectors of FGF signaling, maintain the pluripotency of human stem cells [13] and NC cells [14], and are inhibited during human stem cell differentiation [15]. FGF signaling dysregulation leads to various birth defects, including craniofacial abnormalities and craniosynostosis, such as FGFR2 gain‐of‐function mutations in Apert and Crouzon syndromes [16, 17], which are often accompanied by an open frontal suture [18]. Studies suggest that SMC abnormalities resulting from dysregulated FGF signaling contribute to craniosynostosis [19]. However, the mechanisms by which FGF signaling regulates the formation and function of NC‐derived SMCs remain poorly understood and largely unexplored.

Yap and Taz are downstream effectors of the highly conserved and fundamental canonical Hippo signaling pathway [20, 21, 22]. Despite pivotal roles in regulating cell proliferation, stemness, survival, and differentiation, emerging evidence also highlights cell/context‐specific effects of Yap/Taz [23, 24, 25, 26]. For example, Yap/Taz were found to be inactive in the mouse blastocyst during early embryogenesis, where they help maintain pluripotency and inhibit differentiation [27, 28]. In contrast, in skin and skeletal muscle, they were highly activated in stem/progenitor cells to promote stemness but became inactive in mature cells [29, 30]. The Hippo‐Yap signaling pathway has recently been revealed as an essential regulator in the NC and its lineage‐derived structures [31, 32, 33, 34]. For example, our recent studies showed that Yap and Taz have functional redundancy in the NC and promote NC‐derived osteogenesis by inhibiting chondrogenesis [33]. However, the roles of the Hippo‐Yap signaling pathway in SMCs remain unknown.

Here, we observed a novel interaction between Yap/Taz and Fgf‐Erk signaling, finding that Erk functions as a noncanonical upstream regulator of Yap/Taz activity. Our data showed that activation of Fgf signaling inhibits osteogenesis of NC‐derived cranial SMCs, while enhancing their stemness and proliferation, which could be reversed by reducing Yap/Taz expression. Additionally, our data indicate that Fgf signaling enhances Yap chromatin engagement and transcriptional activity, thereby modulating lineage‐specific gene programs that govern stemness and osteogenesis. Furthermore, we found that YAP directly interacted with phosphorylated ERK1/2 (pERK1/2) and was phosphorylated by pERK1/2 at S128 to promote nuclear translocation. Taken together, our study uncovers a novel molecular mechanism where YAP mediates FGF signaling, enhancing our understanding of stem cell regulation, opening new avenues for exploring diagnostic and therapeutic methods for craniofacial‐related diseases.

## 2 Results

### 2.1 Ectopic Fgf Signaling Causes Expanded Frontal Sutures and a Defective Frontal Bone

**FIGURE 1:**
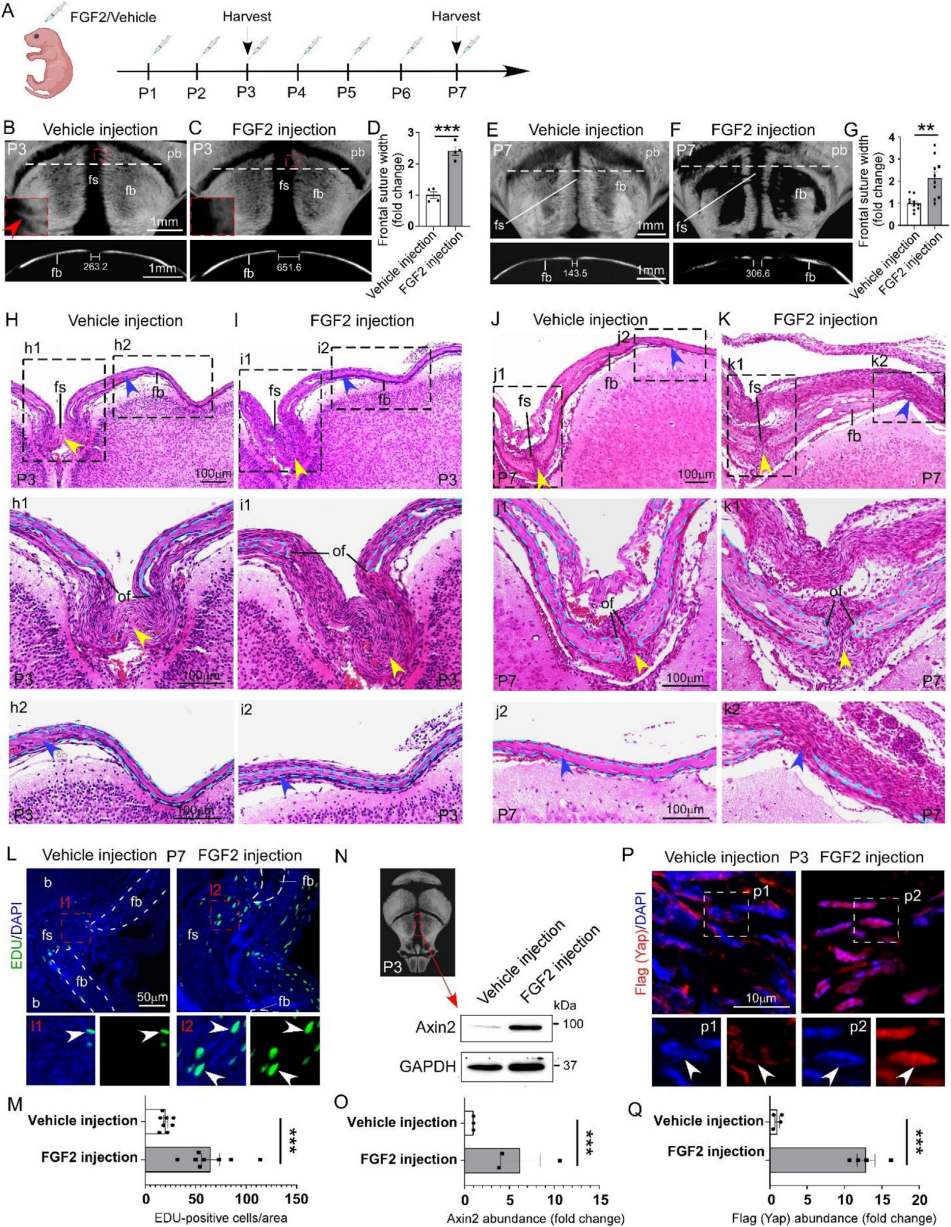
Activation of FGF signaling in SMCs causes a broadened frontal suture and a defective frontal bone in vivo. (A) The vehicle or FGF2 is injected daily into the frontal suture region of neonatal pups from postnatal day 1 (P1) to P7. The head samples are harvested at either P3 or P7. (B‐G) Micro‐computed tomography (µCT) images of the frontal bone and suture regions of the skulls from P3 (B and C) and P7 (E and F) mouse pups with vehicle (B and E) or FGF2 (C and F) injection. White dashed lines indicate the regions shown in the below µCT sections. Red dashed boxes indicate the regions shown in the higher‐magnification images in the bottom left corner. The quantifications show the relative width of the frontal suture in FGF2‐injected pups compared to vehicle‐injected pups at P3 (D) and P7 (G). The red arrowhead indicates the bone growth protrusions of the frontal suture (B), which is absent in FGF2‐injected pups (C). *n* = 4 pups per group for P3 and *n* = 10 pups per group for P7. Scale bar, 1 mm. (H‐K) Hematoxylin and eosin (H&E) staining of coronal sections from the frontal bones and suture regions of P3 (H and I) and P7 (J and K) pups with vehicle (H and J) or FGF2 (I and K) injection. Black boxes indicate the regions shown in the higher‐magnification images below (h1‐k2). Yellow arrowheads point to the frontal suture regions, and blue arrowheads point to normal frontal bones in vehicle‐injected control pups (h2 and j2) and abnormal frontal bones in FGF2‐injected pups (i2 and k2). Blue dashed lines outline the frontal bones. *n* = 4 pups per group for P3, and *n* = 4 pups per group for P7. Scale bar, 100 µm. (L‐M) 5‐ethynyl‐2′‐deoxyuridine (EDU, green) staining (L) and quantification (M) of frontal suture tissue from a coronal section of P7 vehicle‐ (left) or FGF2‐injected (right) pups. Sections are counterstained with DAPI (blue). Red dashed boxes indicate the frontal suture regions shown in the bottom adjacent‐below higher‐magnification images (l1 and l2). White arrowheads point to the EDU‐positive cells. *n* = 4 pups per group. Scale bar, 50 µm (low magnification) and 25 µm (high magnification). (N, O) Western blot (N) and quantification (O) of Axin2 in frontal suture tissues from P3 vehicle‐ and FGF2‐injected mouse pups. GAPDH is used as a loading control. The red dashed box indicates the frontal suture tissue used for western blot. *n* = 3 vehicle‐injected pups and *n* = 5 FGF2‐injected pups. (P, Q) Immunofluorescence (IF) staining (P) and quantification (Q) for Yap with Flag‐Myc‐Tag (Yap, red) expression in the frontal suture from coronal sections of P7 vehicle‐ (left) or FGF2‐injected (right) mouse pups. Sections are counterstained with DAPI (blue). White dashed boxes indicate the regions shown in the adjacent higher‐magnification images below (p1 and p2). White arrowheads point to the localization of the Flag in SMCs. *n* = 4 pups for both vehicle‐ and FGF2‐injected groups. Scale bar, 10 µm (low magnification) and 5 µm (high magnification). Quantitative data are shown as the means ± SEM. Data are expressed as per area changes or the fold change relative to vehicle‐injected pups. An unpaired t‐test is used for quantification. **p < 0.01 and ***p < 0.001. b, brain; fb, frontal bone; fs, frontal suture; of, osteogenic front; pb, parietal bone.

Gain‐of‐function mutations in FGF signaling are widely associated with craniofacial abnormalities due to causing cranial suture defects [35]. Yet, it remains unclear what cellular and molecular changes are caused by the activation of ectopic FGF signaling. To mimic ectopic Fgf signaling in mouse suture, we subcutaneously injected FGF2, a key FGF signaling ligand essential in cranial suture development, into the frontal suture regions of neonatal mouse pups from postnatal day 1 (P1) and harvested samples at P3 and P7 with 5‐ethynyl‐2′‐deoxyuridine (EDU) injection 1 h before harvest (Figure 1A), with controls being littermates receiving vehicle injections in the same regions. Micro‐computed tomography (µCT) data revealed that, compared to control mice, FGF2‐treated mice exhibited significantly widened frontal sutures at both P3 and P7 (Figure 1B–G). Importantly, this phenotype mirrors the frontal suture defects observed in FGFR2 gain‐of‐function mutant mice, such as those with a S252W mutation in *FGFR2* [36, 37]. Notably, we observed severely disturbed ossification and mineralization in the frontal bones of P7 FGF2‐injected pups (Figure 1F) compared to controls (Figure 1E). Although the P3 FGF2‐injected pups did not show obvious frontal bone defects (Figure 1C), they lacked the bone growth protrusions of the frontal suture observed in the control pups (Figure 1B, red arrowhead). Consistently, hematoxylin and eosin (H&E) staining further revealed a significantly widened frontal suture in both P3 (Figure 1I_i1, yellow arrowhead) and P7 (Figure 1K_k1, yellow arrowhead) pups after FGF2 injection compared to controls (Figure 1H_h1,J_j1). We also observed reduced frontal bone formation in P3 (Figure 1I_i2, blue arrowhead) and P7 (Figure 1K_k2, blue arrowhead) in FGF2‐treated pups as compared to control littermates (Figure 1H_h2,J_j2). These data indicated that the activation of ectopic Fgf signaling by FGF2 causes frontal suture overgrowth while repressing ossification and mineralization of the NC‐derived frontal bone.

### 2.2 Fgf Signaling Promotes SMC Proliferation and Stemness

In agreement with the observations of a widened frontal suture and reduced frontal bone ossification and mineralization mentioned above, we found significantly increased cell proliferation, evidenced by the proliferation marker EDU, detected in the FGF2‐injected frontal suture region of P7 pups compared with the same area of controls (Figure 1L,M). Additionally, western blot data from frontal suture tissue identified a remarkably elevated expression of the suture stem cell marker Axin2 in the P3 pups injected with FGF2 compared to controls (Figure 1N,O), suggesting that activation of Fgf signaling enhances the stemness of SMCs. *Yap*‐Tag mice have a fused Myc and Flag tag inserted at the end of the *Yap* gene, to label endogenous *Yap* expression. Using *Yap*‐Tag mice, we observed that FGF2 injection caused a significant increase in nuclear *Yap* expression in the frontal suture of P3 pups compared to controls, as revealed by Yap with Flag‐Myc‐Tag immunofluorescence (IF) staining and quantification (Figure 1P,Q), suggesting a potential crosstalk between Fgf and Yap. These data suggested that ectopic Fgf signaling activated nuclear Yap translocation, promoting proliferation and stemness of the NC‐derived frontal suture.

### 2.3 Fgf Signaling Inhibits Osteogenesis in NC‐Derived SMCs via Yap/Taz

**FIGURE 2:**
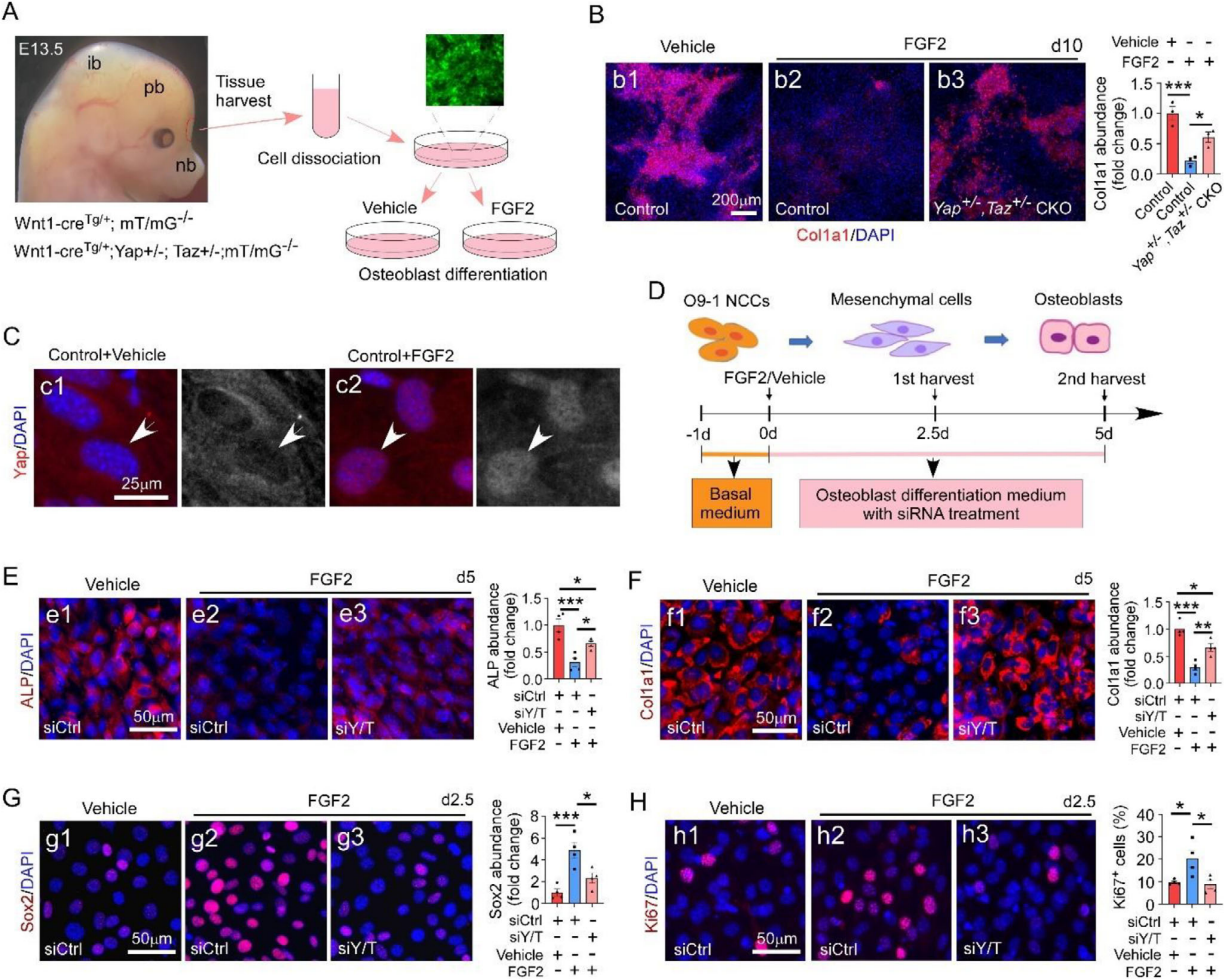
FGF signaling inhibits osteogenesis while promoting stemness and proliferation through Yap and Taz in NC‐derived mesenchymal cells. (A) A schematic showing the isolation and processing of the primary NC‐derived SMCs from the frontal suture of E13.5 mouse embryos. The NC cell lineage is indicated by GFP expression (green). (B) IF staining and quantification of Col1a1 (red) in isolated primary cells from E13.5 control (b1 and b2) and *Yap^+/−^, Taz^+/−^* CKO (b3) mouse embryos after 10 days (d10) of osteoblast differentiation induction following vehicle or FGF2 treatment as indicated. Cells are counterstained with DAPI (blue). *n* = 3 independent experiments with two different fields analyzed in each independent experiment. Scale bars, 200 µm. (C) IF staining showing Yap (red) expression and localization in the isolated primary cells from control embryos with vehicle (c1) or FGF2 (c2) treatment (arrows indicate Yap localization in cells). Cells are counterstained with DAPI (blue). *n* = 3 independent experiments. Scale bars, 25 µm. (D) Strategy used to study the cooperative functions of FGF signaling and Yap/Taz in O9‐1 NC cell osteogenesis (d2.5 and d5). (E, F) IF staining and quantification of ALP (E, red) and Col1a1 (F, red) in O9‐1 NC cells at d5 with indicated conditions. Cells are counterstained with DAPI (blue). n = 4 independent experiments with two different fields analyzed in each independent experiment. Scale bars, 50 µm. (G, H) IF staining and quantification of Sox2 (G, red) and Ki67 (H, red) in O9‐1 NC cells at d2.5 with indicated conditions. Cells are counterstained with DAPI (blue). *n* = 4 independent experiments with two different fields analyzed in each independent experiment. Scale bars, 50 µm. Data represents mean ± SEM, one‐way analysis of variance (ANOVA) combined with Tukey's multiple comparisons test, *p < 0.05, **p < 0.01, ***p < 0.001. ib, interparietal bone; pb, parietal bone; nb, nose bone.

To further explore Yap/Taz‐mediated Fgf signaling in NC‐derived SMCs, we utilized mice with the NC‐specific Cre driver Wnt1‐Cre together with the mTmG reporter. We isolated SMCs from the NC‐derived frontal suture portion of E13.5 control and *Yap/Taz* double heterozygous conditional knockout (*Yap^+/−^, Taz^+/−^ CKO*) mouse embryos, which can be visualized by GFP (Figure 2A). Isolated SMCs were treated with FGF2 to activate Fgf signaling in the experimental groups, while the control groups were treated with the vehicle, and all SMCs were cultured under osteoblast differentiation conditions (Figure 2A). After 10 days of culture, control cells robustly produced the osteogenic marker Col1a1 (Figure 2B_b1), whereas Col1a1 expression was dramatically reduced in FGF2‐treated cells (Figure 2B_b2), suggesting an inhibitory effect of Fgf signaling in osteogenesis. Remarkably, this inhibitory effect was partially counteracted in *Yap^+/−^, Taz^+/−^* CKO cells (Figure 2B_b3), indicating that the adverse effects of Fgf signaling on osteogenesis potentially act through Yap and Taz. Together with these observations, we saw a significant increase in Yap nuclear translocation of FGF2‐treated primary cells compared to controls (Figure 2C). This ex vivo data, combined with our in vivo findings, hints at the possibility that the activation of Fgf signaling inhibits osteogenesis of NC‐derived SMCs by promoting Yap nuclear translocation.

To corroborate these findings in vitro, we used O9‐1 cells, a multipotent NC cell population originally isolated from E8.5 mouse embryos with the capacity to differentiate into multiple cell types under defined culture conditions [38]. During osteogenic induction, NC cells first undergo an intermediate state called NC‐derived mesenchymal cells, then further differentiate into osteoblasts. To manipulate the level of Fgf signaling, we treated O9‐1 cells with FGF2 or a vehicle. Together with culturing O9‐1 cells using osteoblast differentiation media, we performed knockdown (KD) studies using a small interfering RNA (siRNA) smart pool targeting a scrambled siRNA (siCtrl) or both *Yap* and *Taz* (siY/T) (Figure 2D). *Yap* and *Taz* transcription levels were significantly reduced in *Yap/Taz* double knockdown (dKD) cells compared with control cells (Figure S1A). Analogous to our in vivo and ex vivo results, compared to vehicle‐treated control cells after 5 days (d5) of osteogenic induction (Figure 2E_e1, 2 and F_f1, 2), osteogenesis was significantly inhibited after FGF2 treatment, as evidenced by the reduced level of the osteogenic markers ALP (Figure 2E) and Col1a1 (Figure 2F). Respectively, this inhibitory effect was significantly reversed following siY/T treatment (Figure 2E_e3,F_f3). Furthermore, we found a significant increase in the nuclear translocation of Yap in FGF2‐treated O9‐1 NC cells compared with vehicle‐treated controls (Figure S1B). These findings also suggest that activation of Fgf signaling inhibits NC‐derived osteogenesis through Yap/Taz.

### 2.4 Fgf Signaling Promotes Stemness and Proliferation of NC Cells via Yap/Taz

Previous studies showed that expression of Sox2, a key stemness transcription factor that maintains pluripotency and self‐renewal in embryonic stem cells, can be induced by FGF signaling in immature osteoblasts/osteoprogenitors to stimulate osteoblast self‐renewal and inhibit differentiation [39, 40]. We found that during NC‐derived osteogenesis, consistent with decreased osteoblast differentiation, Sox2 was significantly increased in FGF2‐treated cells compared with controls during the early stage (d2.5) of osteogenesis (Figure 2G_g1, 2). Following siY/T treatment, the increased level of Sox2 induced by FGF2 was markedly reversed (Figure 2G_g3), with a concurrent increase of the proliferation marker Ki67 in FGF2‐treated cells as compared to controls (Figure 2H_h1, 2), which was counteracted after siY/T treatment (Figure 2H_h3). These data demonstrate that Fgf signaling promotes the stemness and proliferation of NC cells through Yap/Taz.

These findings align with our in vivo and ex vivo data, unambiguously demonstrating that Fgf signaling inhibits osteogenesis of NC‐derived SMCs while promoting their stemness and proliferation, potentially by increasing Yap nuclear translocation. Together, our data emphasize that the crosstalk between Fgf signaling and Yap/Taz is crucial in orchestrating the balance between osteogenesis and stemness in NC‐derived SMCs.

### 2.5 Yap/Taz‐Mediated FGF Regulation Is Dosage and Stage‐Dependent

To further decipher the roles of Yap/Taz‐mediated FGF regulation of stemness, proliferation, and osteogenesis of NC cells, we utilized *Yap* knockout [33] with *Taz* knockdown (*Yap KO*‐siT) O9‐1 NC cells to completely delete *Yap* and significantly reduce *Taz* expression levels in NC cells (Figure S1C). These cells were cultured under osteoblast differentiation medium and treated with FGF2 or vehicle. We found that the robust level of the osteogenic markers Runx2 and Col1a1 in control cells (Figure S1D_d1 and E_e1) was significantly reduced with FGF2‐treatment (Figure S1D_d2 and E_e2) at d5 osteogenesis. However, FGF2‐mediated inhibition of osteogenesis was not restored in *Yap KO*‐siT cells (Figure S1D_d3 and E_e3). In contrast to the restoration observed in siY/T‐treated cells (Figure 2E_e3,F_f3), Runx2 and Col1a1 levels in FGF2‐treated *Yap KO*‐siT cells were comparable to, or slightly lower than, FGF2‐treated control cells (Figure S1D_d3 and E_e3). These findings indicate the requirement of Yap in NC osteogenesis, and demonstrate that excessively low expression levels of Yap/Taz also diminish osteogenesis.

Staining of the stemness marker Sox2 showed that Sox2 levels gradually decreased in O9‐1 NC cells under osteogenic differentiation culture conditions [33]. Compared to the high level of Sox2 in FGF2‐treated control cells (Figure S1F_f2) at d2.5 osteogenesis, the level of Sox2 in FGF2‐treated *Yap KO*‐siT cells (Figure S1F_f3) was significantly reduced and comparable to vehicle‐treated control cells (Figure S1F_f1). This data indicates that cell stemness correlates positively with Yap/Taz expression levels, and that Fgf signaling promotes stemness of NC cells via Yap/Taz. These findings reveal a critical interaction between Fgf signaling and Yap/Taz that must be properly fine‐tuned for regulating the stemness and osteogenesis of NC cells.

### 2.6 Altered Yap Targets in NC‐Derived SMCs of *Fgfr2^+/^* ^S252W^ Mouse Embryos

Our FGF2‐treated mice exhibited abnormally widened frontal sutures (Figure 1C), mirroring the frontal suture phenotype observed in *Fgfr2^+/^* ^S252W^ mice with the FGF signaling gain‐of‐function mutation [41]. S252W is the most common FGF gain‐of‐function mutation occurring in two‐thirds of patients with Apert syndrome, characterized by abnormal craniofacial and cranial suture development [16]. Accordingly, we tested whether our hypothesis, regarding the coordination between Fgf and Hippo‐Yap signaling, applies to the Apert syndrome *Fgfr2^+/^* ^S252W^ mouse. Intriguingly, by overlaying potential Yap targets identified in CUT&RUN data from O9‐1 NC cells [33] with differentially expressed genes (DEGs) identified in published bulk RNA‐seq datasets from SMCs of the NC‐derived frontal suture from E18.5 wildtype (wt) and *Fgfr2^+/^* ^S252W^ mice [37], we observed that over 50% of DEGs in SMCs of *Fgfr2^+/^* ^S252W^ mice were potential Yap targets (Figure S2A). Gene Ontology (GO) term analysis revealed that many of these overlapping genes are involved in regulating cell proliferation and osteogenesis. Among these overlapping DEGs, many of the upregulated genes encoded products associated with positive regulation of the cell cycle and negative regulation of osteoblast differentiation (Figure S2B), including the known Yap targets *Cdk4* and *Ccn2* (Figure S2C and D). Notably, transcripts related to the FGF signaling downstream pathway MAPK were indicated in the top‐enriched GO terms (Figure S2B). The MAPK‐ERK pathway is one of the primary downstream sub‐pathway of FGF signaling and has a vital role in cranial suture development [19]. Many of the down‐regulated genes encoded products related to extracellular matrix organization and ossification (Figure S2E). These data further suggest that the FGFR2 gain‐of‐function mutation may affect the proliferation and osteogenesis of NC‐derived SMCs by modulating the transcription of Yap target genes, leading to a widened frontal suture.

### 2.7 FGF‐Regulated Yap Activity Is Mediated by pERK1/2

**FIGURE 3:**
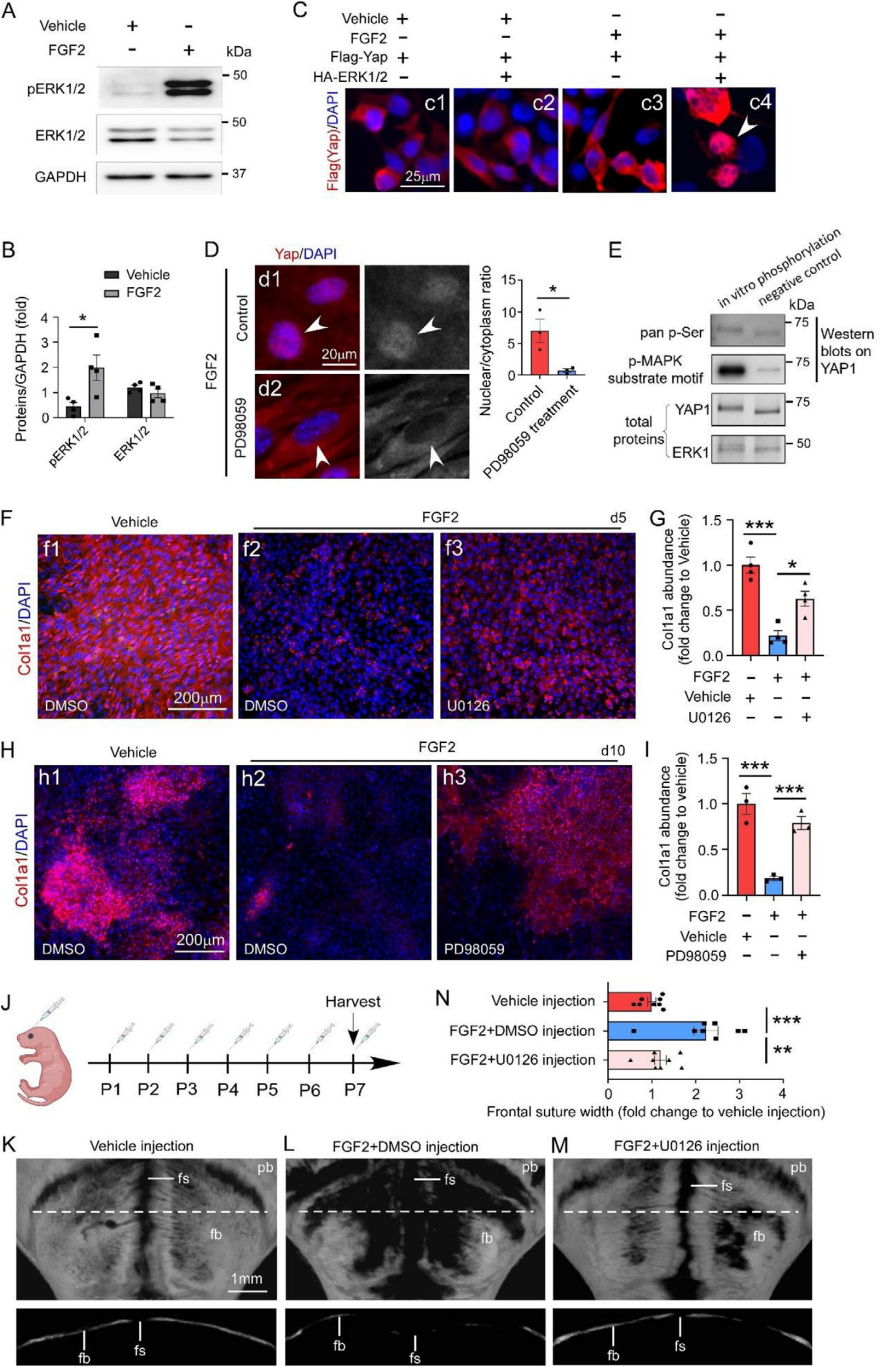
pERK1/2 repression mitigates the functions of FGF signaling. (A, B) Western blot for ERK1/2 and pERK1/2 expression (A) and their quantification (B) in O9‐1 NC cells after d2.5 osteogenic induction, following vehicle or FGF2 treatment. *n* = 4 independent experiments. (C) Tag indicated Yap(red) expression in 293T cells transfected with Flag‐Yap and/or HA‐ERK1/2 plasmid, following vehicle or FGF2 treatment as indicated. Cells were counterstained with DAPI (blue). The white arrowhead points to the nuclear enrichment of Yap (c4). *n* = 4 independent experiments. Scale bar, 25 µm. (D) Yap (red) expression pattern and quantification in NC‐derived SMCs isolated from the frontal suture region of E13.5 mouse embryos, following FGF2 and/or pERK1/2 inhibitor PD98059 treatment. Cells were counterstained with DAPI (blue). White arrowheads point to the localization of Flag (Yap). *n* = 3 independent experiments. Scale bar, 20 µm. (E) Phosphorylation assay indicated a direct phosphorylation of YAP by ERK kinase activity. Negative control is the EDTA‐caused quenched kinase activity. (F, G) Col1a1 (red) expression (F) and quantification (G) in d5 osteogenic induced O9‐1 NC cells, following treatment with vehicle (f1) or FGF2 without (f2) or with (f3) the pERK1/2 inhibitor U0126. Cells are counterstained with DAPI (blue). Scale bar, 200 µm. (H, I) Col1a1 (red) expression (H) and quantification (I) in d10 osteogenic induced NC‐derived SMCs isolated from the frontal suture region of E13.5 mouse embryos, following treatment with vehicle (h1) or FGF2 without (h2) or with (h3) pERK1/2 inhibitor PD98059. Cells are counterstained with DAPI (blue). Scale bar, 200 µm. (J) Schematic depicting either vehicle or FGF2 daily frontal suture region injections of neonatal pups from postnatal day 1 (P1) to P7. The head samples are harvested at P7. (K–N) µCT images of the frontal bone and suture regions of skulls from P7 mouse pups with vehicle (k1), FGF2 (k2), or FGF2 together with U0126 (k3) injection. White dashed lines point out the position of the below µCT section images. Quantification shows the relative width of the frontal suture in FGF2‐ and/or U0126‐injected pups compared to vehicle‐injected pups (N). *n* = 8 pups per group. Scale bar, 1 mm. Data represents mean ± SEM. The unpaired t‐test is used to quantify B, D. ANOVA combined with Tukey's multiple comparisons test was used for G, I, and N. *p < 0.05, **p < 0.01, and ***p < 0.001. fb, frontal bone; fs, frontal suture; pb, parietal bone.

Our data above suggest that the MAPK‐ERK1/2 sub‐pathway of FGF signaling participates in regulating Yap activity in NC‐derived SMCs. To validate this finding, we examined the downstream effectors of Fgf signaling, including pERK1/2 and pAKT in O9‐1 NC cells after d2.5 osteogenic induction with FGF2 or vehicle treatment. We observed that the pERK1/2 was significantly increased in FGF2‐treated cells compared to control cells (Figure 3A,B and Figure S3A), while pAKT did not show a noticeable change (Figure S3B). These findings indicate that FGF2 regulates Yap activity through activated ERK1/2 but not via AKT.

To further investigate whether activated pERK1/2 is required for Yap nuclear translocation, we transfected Flag‐YAP and/or HA‐ERK1/2 into 293T cells, a human cell line derived from HEK293 (Human Embryonic Kidney 293) cells, and treated with either FGF2 or vehicle (Figure 3C). In accordance with our hypothesis, we found that cells with both YAP and ERK1/2 transfection, along with FGF2 treatment showed robust nuclear YAP translocation (Figure 3C_c4), while both FGF2 treatment alone (Figure 3C_c3) or YAP and ERK1/2 co‐transfection without FGF2 treatment (Figure 3C_c2) failed to promote YAP nuclear translocation in cells. These data indicate that FGF2‐activated pERK1/2 plays a critical role in YAP nuclear translocation. To further validate this result, we isolated NC‐derived SMCs from the E13.5 NC‐derived frontal suture (Figure 2A) and treated these cells with PD98059 (Figure 3D), a selective inhibitor of MEK activation, thereby blocking MAP kinase signaling and phosphorylation of ERK1/2 [42]. Consistently, the increased nuclear translocation of YAP, enhanced by FGF2 in these cells (Figure 3D_d1), was strongly repressed by PD98059 (Figure 3D_d2). Importantly, our in vitro phosphorylation assay data indicated that ERK can directly phosphorylate YAP, while this phosphorylation reaction can be terminated by EDTA, causing quenched kinase activity (Figure 3E). These findings supported our conclusion that activated pERK1/2 is required for FGF2‐stimulated YAP nuclear translocation.

### 2.8 pErk1/2 Repression Mitigates the Effects of Fgf Signaling

To gain additional insight into whether the inhibitory role of FGF signaling on osteogenesis of NC‐derived mesenchymal cells could be reversed by pErk1/2 inhibition, we cultured O9‐1 NC cells in osteoblast differentiation medium in the presence or absence of a FGF2 ligand and the pErk1/2 inhibitor U0126 (Figure 3F). Similar to PD98059, U0126 specifically inhibits the Erk1/2 upstream activator Mek1/2 to repress Erk1/2 phosphorylation [43]. Consistent with previous data, osteogenesis was significantly inhibited in FGF2‐treated cells (Figure 3F_f2) compared with vehicle‐treated controls (Figure 3F_f1,G). The inhibitory functions of FGF2 on osteogenesis of O9‐1 NC cells were significantly blocked by U0126, as evidenced by an increase in the level of the osteogenesis marker Col1a1 in U0126 and FGF2 double treatment cells (Figure 3F_f3), in comparison to FGF2 treatment alone (Figure 3F_f2). Next, we used isolated frontal SMCs from the frontal suture region of E13.5 mouse embryos to further validate this conclusion. In line with our in vitro findings, FGF2 significantly repressed osteogenesis of these isolated cells compared with controls (Figure 3H_h1, h2, and I), and this repression was significantly blocked by the pERK1/2 inhibitor PD98059 (Figure 3H_h3 and I).

To further validate this result in vivo, we subcutaneously injected FGF2 and/or the pErk1/2 inhibitor U0126 into the frontal suture regions of neonatal mouse pups to modulate pErk1/2 activities in frontal SMCs from P1 to P7 (Figure 3J). Meanwhile, vehicle injections into the same areas of littermates were used as controls. All head samples were harvested at P7. µCT data revealed that, compared to vehicle‐injected control pups (Figure 3K), the abnormal widening of the frontal suture and reduced frontal bone mineralization caused by FGF2 ectopic activation in FGF2‐injected pups (Figure 3L,N) were significantly rescued by U0126‐injection (Figure 3 M,N). Collectively, our data further support the conclusion that Fgf signaling represses osteogenesis of NC‐derived mesenchymal cells by modulating Yap/Taz via pErk1/2.

### 2.9 FGF Signaling Promotes ERK1/2 and Yap Interaction

**FIGURE 4:**
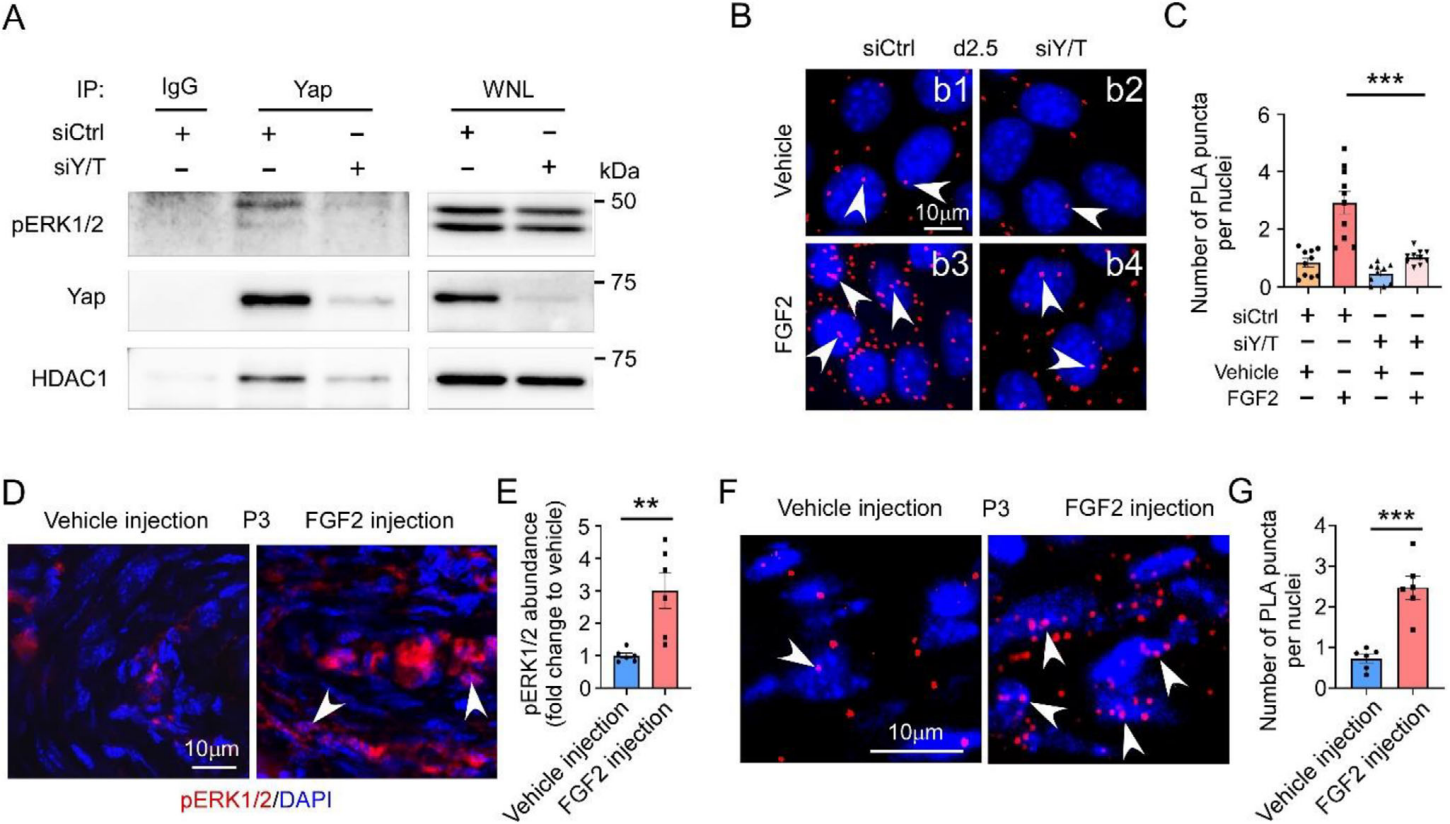
Fgf signaling enhances Yap nuclear translocation by promoting the interaction of pErk1/2 and Yap. (A) Co‐immunoprecipitation (Co‐IP) for pErk1/2 and Yap in Yap nuclear immunoprecipitants of O9‐1‐NC cells treated with siCtrl or siY/T. IgG immunoprecipitation is a negative control. Western blot is used to check the expression of pErk1/2 and Yap in whole nuclear lysates (WNL) of O9‐1 NC cells. HDAC1 is a nuclear protein loading control. *n* = 3 independent experiments. (B, C) In situ proximity ligation assay (PLA) for pErk1/2 and Yap in siCtrl and siY/T O9‐1 NC cells after d2.5 osteogenic induction, following vehicle or FGF2 treatment (B), as well as the quantification of the number of PLA puncta per nuclei (C). Cells were counterstained with DAPI (blue). White arrowheads point to the interaction spots of pErk1/2 and Yap in the nuclei. *n* = 3 independent experiments. Scale bar, 10 µm. (D, E) pErk1/2 (red) expression (D) and quantification (E) in SMCs from frontal suture tissues of P3 mouse pups, following vehicle (left) or FGF2 (right) injection. Cells were counterstained with DAPI (blue). White arrowheads point to the high nuclear expression of pErk1/2. *n* = 3 mouse pups. Scale bar, 10 µm. (F, G) PLA for pErk1/2 and Yap in SMCs of frontal suture tissues from P3 mouse pups, following vehicle (left) or FGF2 (right) injection (F), as well as the quantification of the number of PLA puncta per nuclei (G). Cells were counterstained with DAPI (blue). Nuclei were outlined by white dashed lines. White arrowheads point to the interaction spots of pErk1/2 and Yap in the nuclei. *n* = 3 mouse pups. Scale bar, 5 µm. Data represents mean ± SEM. ANOVA combined with Tukey's multiple comparisons test was used for C. The unpaired t‐test is used for quantifying E and G. **p < 0.01 and ***p < 0.001.

Our nuclear co‐immunoprecipitation (co‐IP) of O9‐1 NC cells by anti‐Yap antibody revealed a physical interaction of Yap with pErk1/2 in control cells as compared to siY/T‐treated cells (Figure 4A). A similar interaction between YAP and ERK1/2 was also observed in 293T cells transfected with human Flag‐YAP and HA‐ERK1/2 plasmids (Figure S3C). We then conducted proximity ligation assay (PLA) in O9‐1 NC cells under osteogenic induction for 2.5 days (d2.5) with FGF2 or vehicle treatment to further evaluate the interaction and location of Yap and pErk1/2, while also monitoring how FGF2 mediates this interaction during osteogenesis (Figure 4B,C). Our results showed that the interaction between Yap and pErk1/2 was significantly increased in the nucleus of FGF2‐treated cells, as indicated by the detection of PLA signal (red spots, pointed by white arrowhead) (Figure 4B_b3) compared to the controls (Figure 4B_b1), and their interaction in the nucleus was notably reduced in siY/T cells (Figure 4B_b2, 4). This implies that FGF2 enhances Yap nuclear translocation by promoting the interaction between Yap and pErk1/2. In agreement with this observation, we observed significantly increased pErk1/2 levels (Figure 4D,E) and the amount of interactions between Yap and pErk1/2 (Figure 4F,G) in NC‐derived frontal SMCs of FGF2‐injected P3 mice as compared to those of vehicle‐injected controls.

### 2.10 Conserved FGF‐YAP Regulation in Human Cells

**FIGURE 5:**
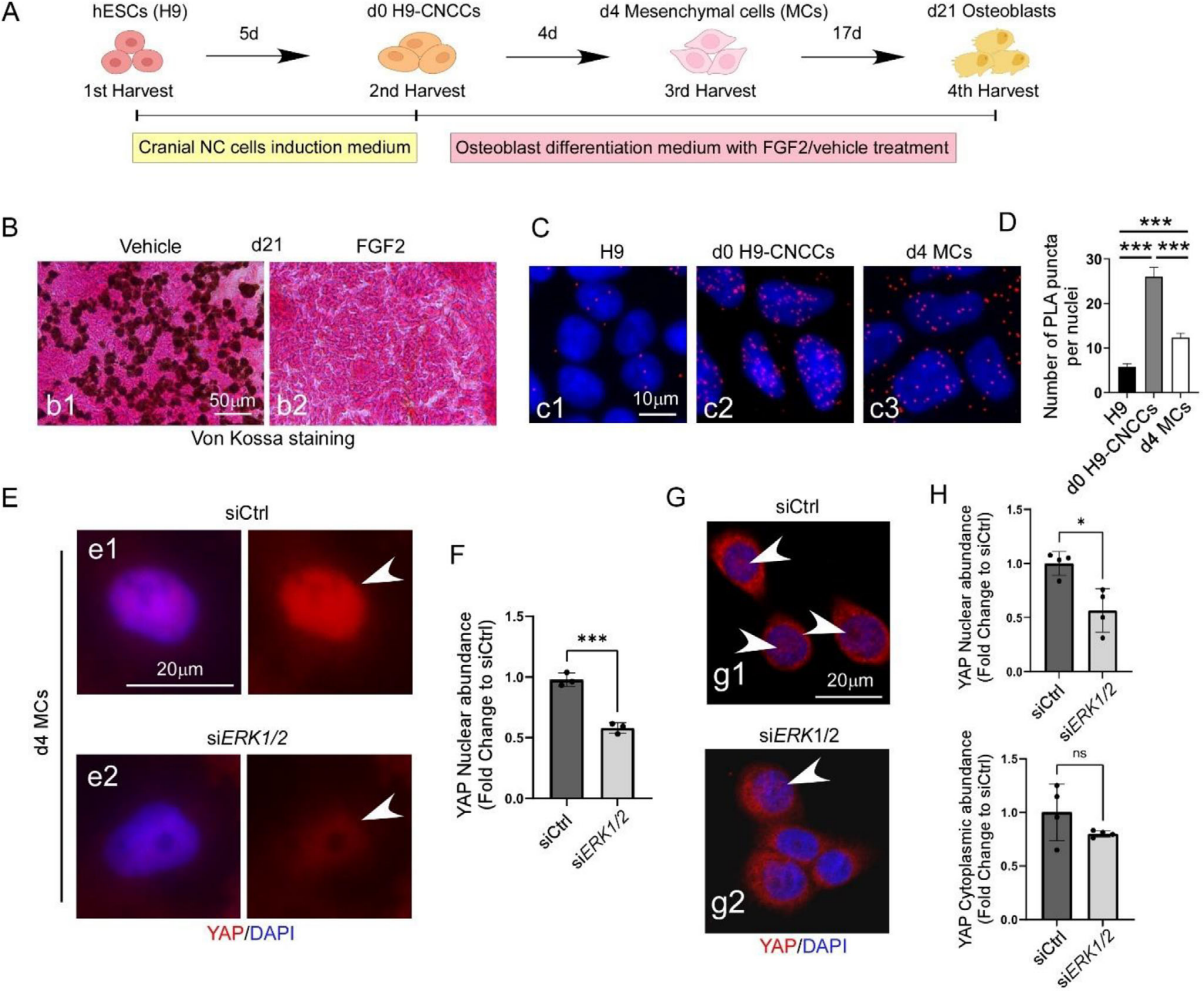
FGF‐YAP regulation is conserved in human cells. (A) Schematic shows the process of differentiating H9 human embryonic stem cells (hESCs) into cranial NC cells using cranial NC cells induction medium, followed by their further differentiation into osteoblasts using osteoblast differentiation medium, with vehicle or FGF2 treatment, to assess the effect of FGF signaling on osteogenesis. (B) Von Kossa staining of H9‐derived cranial NC cells (H9‐CNCCs) after d21 of inducing osteogenic differentiation, following treatment with vehicle (b1) or FGF2 (b2). Scale bar, 50 µm. (C, D) PLA for pERK1/2 and YAP in H9 hESCs (c1), H9‐CNCCs (c2), and d4 osteogenic induced H9‐CNCC‐derived mesenchymal cells (d4 MCs) (c3), as well as the quantification of the number of PLA puncta per nuclei (D). (E, F) FGF‐induced nuclear YAP levels were significantly repressed in 4d MCs treated with si*ERK1/2* knockdown (e2) compared to treatment with control siRNA (e1), which is also indicated by the quantification of YAP nuclear intensity (F). *n* = 3 independent experiments, with four different fields analyzed. (G, H) IF staining of HaCaT cells under FGF2 treatment showed a significant reduction in nuclear YAP localization, compared siCtrl treatment (g1) and si*ERK1/2* treatment (g2) comparing siCtrl treatment (g1), si*ERK1/2* treatment reduced YAP nuclear activity (g2), followed by both nuclear and cytoplasmic YAP quantification (H). Cells were counterstained with DAPI (blue). The white arrowheads point to the nuclear enrichment of YAP. *n* = 4 independent experiments. Scale bar as indicated. Data represent mean ± SEM. ANOVA combined with Tukey's multiple comparisons test for quantifications of D. The unpaired t‐test is used for quantifying F and H. **p < 0.01 and ***p < 0.001.

To unravel the interaction between YAP and pERK1/2 and provide insight into a potential role in the osteogenic process of human NC cells, we differentiated H9 Human embryonic stem cells (hESCs) into cranial NC cells and then cultured them in osteoblast differentiation medium to induce osteogenesis, with either FGF2 or vehicle treatment (Figure 5A). These cells were harvested at various times, as indicated in Figure 5A. Accordingly, we assessed the impact of ectopic activation of FGF signaling on osteoblast differentiation by Von Kossa staining, which quantifies cell mineralization by detecting calcium deposits. Consistently, compared to vehicle‐treated control cells, which exhibited rich calcium deposits (Figure 5B_b1), FGF2‐treated cells did not show any cell mineralization, as indicated by the absence of Von Kossa‐positive‐stained cells (Figure 5B_b2). Additionally, using PLA (Figure 5C and D), we found that the H9‐derived cranial NC cells (H9‐CNCCs) (Figure 5C_c2), but not the undifferentiated H9 cells (Figure 5C_c1), exhibited strong nuclear enrichment of PLA signals, demonstrating a substantially increased interaction between YAP and pERK1/2 in H9‐CNCCs. Notably, this interaction was significantly reduced in the nuclei of H9‐CNCC‐derived mesenchymal cells after 4 days of osteogenic induction (4d MCs) (Figure 5C_c3), suggesting that this interaction may potentially inhibit osteogenesis of cranial NC cells. Additionally, siRNA knockdown of *ERK1/2* in 4d MCs significantly reduced the FGF‐induced nuclear YAP levels (Figure 5E_e2 and F), compared to 4d MCs treated with scrambled siRNA (siCtrl) (Figure 5E_e1). This finding is in line with our mouse ex vivo and in vitro results, indicating that the YAP‐pERK1/2 interaction also occurs in human cranial NC cells and potentially mediates FGF signaling to maintain human cranial NC pluripotency and inhibit their osteogenesis. This conclusion supports the conservation of our findings in human NC cells and NC‐derived mesenchymal cells. To determine if these findings extended beyond NC‐derived mesenchymal cells, we performed siRNA knockdown of *ERK1/2* in HaCaT cells and treated them with FGF2. HaCaT cells are derived from human epidermal keratinocytes and are widely used for investigating toxicology and carcinogenesis [44]. Similar to our findings in NC cells and NC‐derived mesenchymal cells, under FGF2 treatment, HaCaT cells with siERK1/2 showed significantly reduced YAP nuclear localization (Figure 5G_g2,H), compared to HaCaT cells treated with siCtrl (Figure 5G_g1). However, there was no significant change in YAP's cytoplasmic staining in HaCaT cells with reduced ERK1/2 levels (Figure 5H). Together, these data support a conserved FGF‐YAP regulation in human cells.

### 2.11 Fgf Signaling Promotes Transcriptional Regulatory Activity of Yap

**FIGURE 6:**
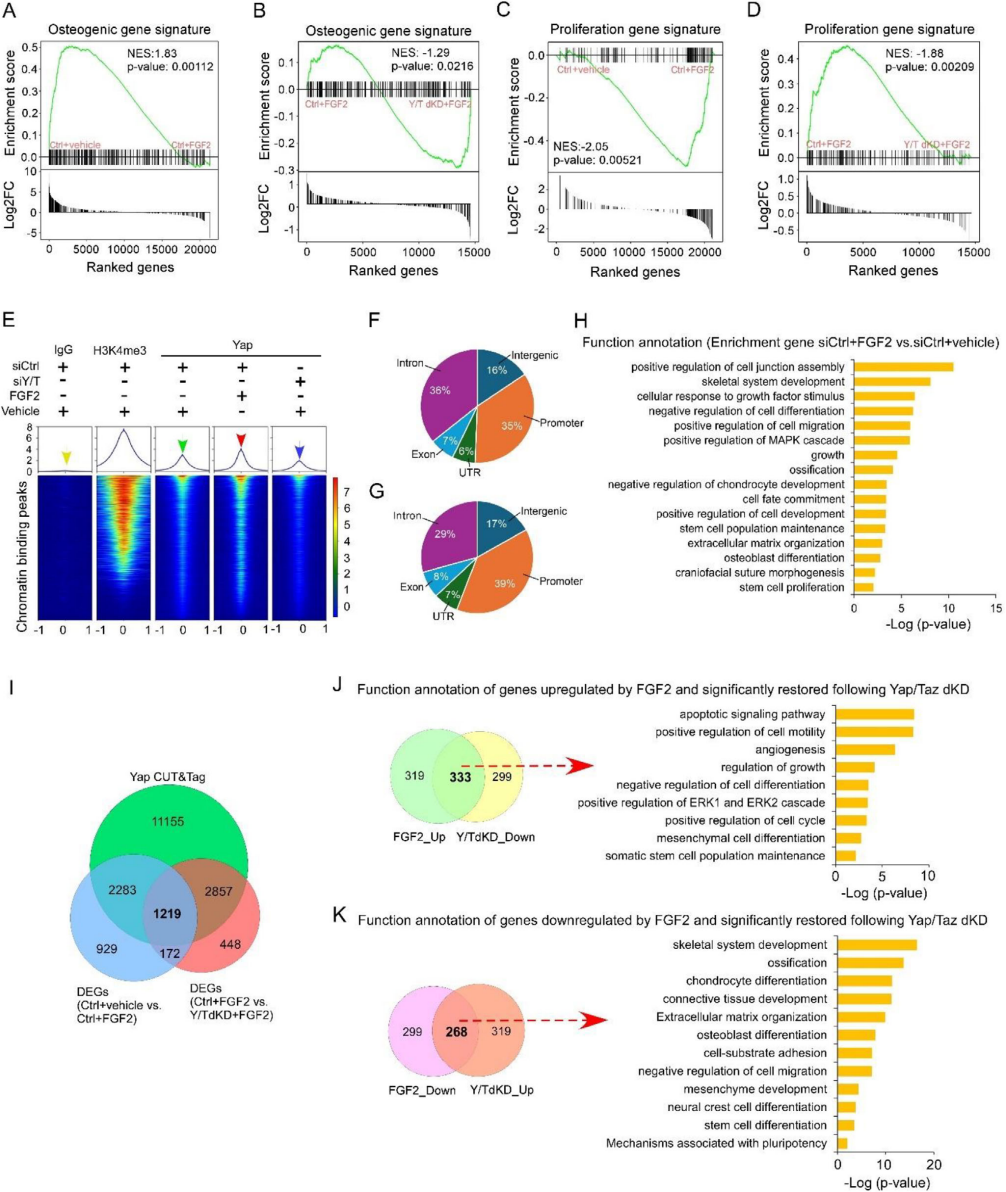
FGF signaling promotes Yap transcriptional regulatory activity. (A–D) Gene set enrichment analysis (GSEA) showing enrichment of the transcriptional signatures for osteogenesis (A and B) and proliferation (C and D) in vehicle‐ and scramble siRNA (siCtrl)‐treated cells (Ctrl + vehicle) compared to FGF2‐ and siCtrl‐treated cells (Ctrl + FGF2) (A and C), as well as FGF2‐ and siCtrl‐treated cells (Ctrl + FGF2), compared to FGF2‐ and Yap/Taz targeted siRNA (si*Y/T*)‐treated cells (Y/T dKD + FGF2) (B and D). (E) CUT&Tag heatmap (below) and histograms of the mean signal for the heatmaps (above) showing IgG, Yap, and H3K4me3 DNA binding peaks in d2.5 osteogenic induction of O9‐1 NC cells following vehicle/FGF2 or siRNA treatment as indicated. The yellow arrow points to IgG binding peaks in siCtrl cells with vehicle treatment. Green, red, and blue arrows point to Yap binding peaks in vehicle‐treated siCtrl cells, FGF2‐treated siCtrl cells, and vehicle‐treated si*Y/T* cells, respectively. (F, G) Distribution of Yap‐associated regions in vehicle‐treated siCtrl cells (F) and FGF2‐treated siCtrl cells (G). UTR, untranslated region. (H) Gene Ontology (GO) analysis of differentially enriched Yap‐bound genes between vehicle‐ and FGF2‐treated siCtrl cells. (I) Venn diagram showing the overlap of Yap‐bound genes (1219 genes) identified from CUT&Tag‐seq (green) with DEGs between the Ctrl+vehicle and Ctrl+FGF2 cells (blue), as well as the DEGs between the Ctrl+FGF2 and Y/T dKD+FGF2 cells (red) identified in the RNA‐seq data from d2.5 osteogenic induced O9‐1 NC cells. (J, K) GO analysis of candidate Yap target genes that are upregulated (J) or downregulated (K) by FGF2 and restored following *Yap/Taz* double knockdown (dKD).

Our findings above indicated that Fgf signaling is critical in balancing osteogenesis and stemness of NC‐derived mesenchymal cells via Yap and Taz. To dissect the molecular mechanism underlying these processes, we used siY/T (*Yap/Taz* dKD) or siCtrl to modulate Yap/Taz expression levels in O9‐1 NC cells cultured in osteoblast differentiation medium with FGF2 or vehicle treatment and collected cells at d2.5 and d5 for bulk RNA‐seq analysis (Figure S4A and B). The gene set enrichment analysis (GSEA) for the d2.5 groups showed that FGF2‐treated cells (Ctrl + FGF2) had a significantly reduced osteogenic gene signature compared to the control cells (Ctrl + vehicle) (Figure 6A), yet the osteogenic gene signature was restored by *Yap/Taz* double knockdown (Y/T dKD + FGF2) (Figure 6B). Conversely, we found FGF2 treatment lead a dramatic enrichment of proliferation gene signatures (Figure 6C) while *Yap/Taz* dKD diminished that enrichment (Figure 6D). Similar results were also found in d5 groups of cells for osteogenesis gene signatures (Figure S4C and D), yet not for proliferation gene signatures (Figure S4E and F), which is likely due to the significant increase in d5 cell density known to influence proliferation [45, 46]. These findings indicate that Fgf signaling represses osteogenic genes while activating proliferation‐related genes through Yap and Taz.

To study how Fgf signaling operates through Yap, we performed cleavage under targets and tagmentation sequencing (CUT&Tag‐seq) using antibodies against Yap1, H3K4me3, and immunoglobulin G (IgG) in O9‐1 NC cells after d2.5 of osteogenic induction with FGF2 or vehicle treatment. H3K4me3, known for its high enrichment at active promoters near transcription start sites and for being positively correlated with transcription [47], was used as a positive control. In addition to IgG, *Yap/Taz* dKD (siY/T) cells were also used as a negative control. Our CUT&Tag‐seq heatmap (Figure 6E) exhibited increased Yap chromatin occupation (red arrow) in FGF2‐treated cells compared to vehicle‐treated controls (green arrow), as indicated by the peaks showing chromatin activity. The peaks were visibly decreased in both IgG (Figure 6E, yellow arrow) and *Yap/Taz* dKD (Figure 6E, blue arrow) negative control cells. These findings suggest that Fgf signaling regulates Yap target genes at a genomic scale by modulating Yap binding on its targets. In control cells, 35% of Yap binding events were localized to promoter regions and 36% to the intronic regions (Figure 6F), while in FGF2‐treated cells, 39% of Yap binding events were localized to promoter regions and 29% to the intronic regions (Figure 6G). GO analysis for the differential enrichment of Yap‐bound genes between control and FGF2‐treated cells revealed that many of the altered genes following FGF2 treatment were involved in skeletal system development, ossification, and osteoblast differentiation, as well as in negative regulation of cell differentiation, stem cell population maintenance, and stem cell proliferation (Figure 6H).

We further overlapped the putative Yap target genes identified by the above CUT&Tag‐seq with the DEGs between the Ctrl and Ctrl+FGF2 cells, as well as the DEGs between the Ctrl+FGF2 and Y/T dKD+FGF2 cells identified in the d2.5 bulk RNA‐seq data (Figure 6I). We found that about 86% of the DEGs regulated by Yap/Taz, identified from the Ctrl+FGF2 and Y/T dKD+FGF2 cells, were shared with the putative Yap target genes identified by CUT&Tag‐seq. Interestingly, more than 76% of the DEGs regulated by Fgf signaling, identified from the Ctrl and Ctrl+FGF2 cells, also overlapped with these putative Yap targets (Figure 6I), supporting our hypothesis that Fgf signaling potentially regulates Yap targets. In addition, about 26% (1219 overlapping genes) of Yap/Taz‐regulated genes, which overlapped with the putative Yap target genes from CUT&Tag‐seq, were also regulated by Fgf signaling (Figure 6I). Strikingly, around 51.1% (333 genes) of these overlapping genes upregulated by Fgf signaling were significantly restored following *Yap/Taz dKD* (Figure 6J). GO term analysis revealed that many of these genes encode products related to stemness and proliferation, including negative regulation of cell differentiation, positive regulation of the cell cycle, and maintenance of somatic stem cell populations (Figure 6J). Meanwhile, 47.3% (268 genes) of the overlapping genes downregulated by Fgf signaling were substantially restored following *Yap/Taz dKD* (Figure 6K). Many of these genes are associated with osteogenesis, such as ossification, osteoblast differentiation, and stem cell differentiation (Figure 6K). Similar results were also observed when we overlapped the putative Yap target genes from CUT&Tag‐seq with DEGs from bulk RNA‐seq data of d5 groups (Figure S4G–I). These findings demonstrate that Yap/Taz mediate the functions of Fgf signaling in multiple cell events and processes, including cell stemness, proliferation, and osteogenesis.

### 2.12 Fgf Signaling Regulates Yap Chromatin Occupation to Control Yap Targets

**FIGURE 7:**
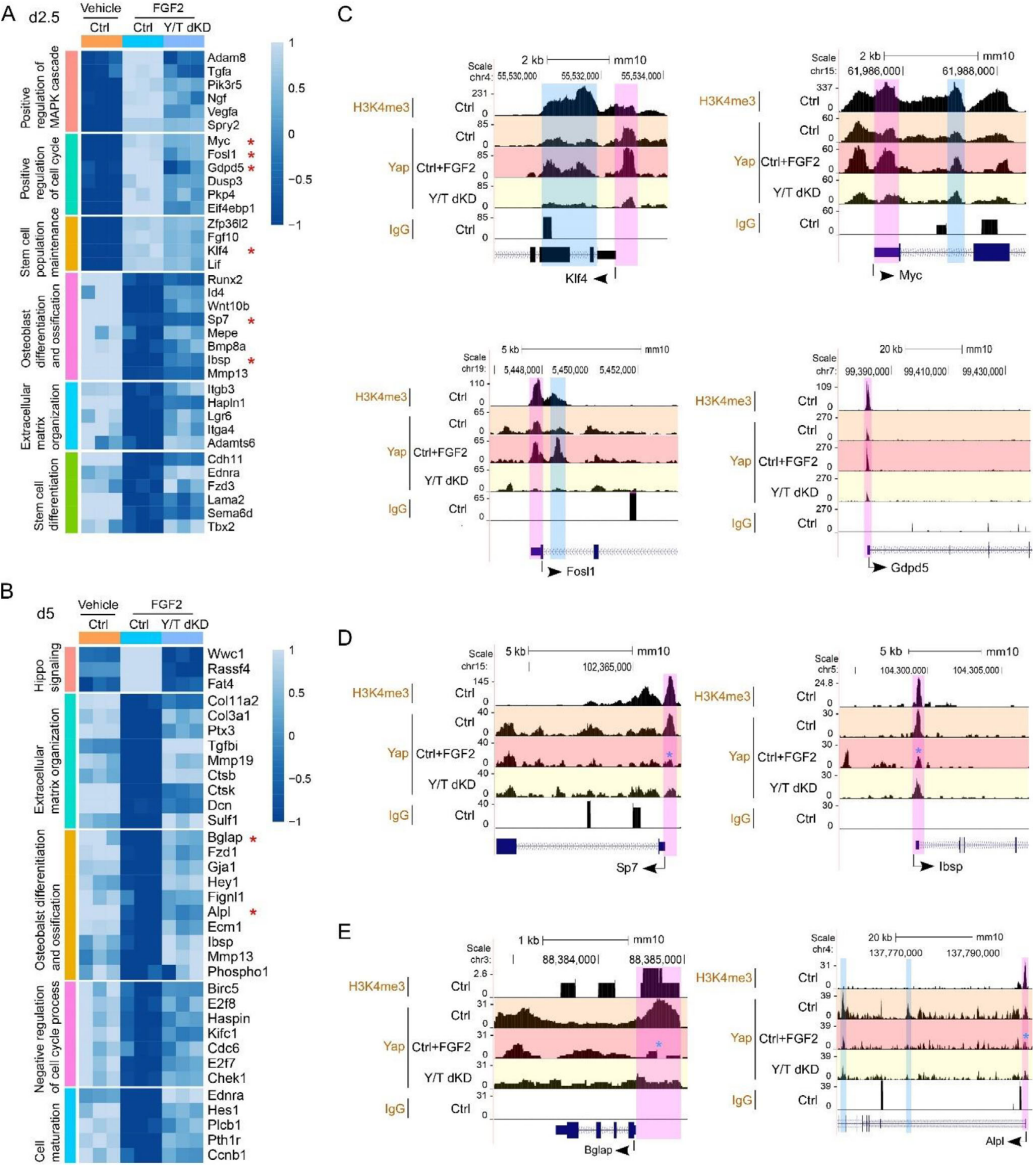
FGF signaling regulates Yap targets by modulating Yap chromatin occupation. (A, B) Heatmap showing the transcripts of representative candidate Yap targets that are differentially increased and decreased in O9‐1 NC cells treated with FGF2 and siCtrl (Ctrl) compared to vehicle‐ and siCtrl‐ treated cells and are restored in si*Y/T*‐treated (Y/T dKD) cells despite FGF2 treatment, at d2.5 (A) and d5 (B) of osteogenic induction. Red asterisks indicate genes of interest, as shown in Figure 7C,D, and E. (C–E) Peak calling from CUT&Tag‐seq for H3K4me3, Yap and IgG at representative stemness (Klf4) and proliferation (Myc, Fosl1, Gdpd5) related genes at d2.5 of osteogenic induction (C), as well as osteogenesis related genes at d2.5 (Sp7 and Ibsp) (D) and d5 (Bglap and Alpl) (E) of osteogenic induction. These representative genes were chosen from heatmaps (A and B, red asterisk). The annotated open chromatin region of the changed intronic region (blue) and promoter region (pink) in these genes is highlighted. Blue asterisks indicate a decreased peak in the FGF2‐treated siCtrl group compared to the vehicle‐treated siCtrl group at the promoter region of the genes.

To further gain mechanistic insight into the regulation of Yap target genes by Fgf signaling, we identified key genes involved in regulating cell stemness, proliferation, and osteogenesis (Figure 7A,B), depending on the GO term analysis (Figure 6J,K, and Figure S4H,I) of both d2.5 and d5 osteogenic induction of NC cells. These genes were coordinately regulated by Fgf signaling and Yap/Taz (Figure 7A,B). Stemness‐ and proliferation‐related genes, such as *Klf4*, *Myc*, *Fosl1*, and *Gdpd5*, displayed a noticeable increase in Yap occupation of their active chromatin regions, particularly the promoter regions (highlighted in red) in the FGF2‐treated cells (Ctrl+FGF2) compared to the control cells (Ctrl) (Figure 7C). In contrast, key osteogenesis genes, such as *Sp7* and *Ibsp* at d2.5 (Figure 7D), as well as *Bglap* and *Alpl* at d5 (Figure 7E), exhibited a striking reduction in Yap occupancy in their promoter regions (highlighted in red with a blue asterisk) in the Ctrl+FGF2 cells compared to the Ctrl cells. As a negative control, a dramatic decrease in Yap occupation in the same chromatin regions of these genes was observed in the Y/T dKD cells (Figure 7C–E). These findings reveal that Fgf signaling regulates Yap targets by either enhancing or repressing Yap's binding to their open chromatin to maintain the balance between self‐renewal and differentiation in NC‐derived mesenchymal cells.

### 2.13 pERK1/2 Phosphorylates YAP at the Serine 128 (S128) Site

The nuclear/cytoplasm translocation of Yap is typically associated with its phosphorylation at serine 127 (pYap S127) by the canonical Hippo kinases Lats1/2, which promotes cytoplasmic retention and degradation via binding of the 14‐3‐3 protein. Therefore, we examined the level of pYap S127 in O9‐1 NC cells to gain further mechanistic insight into how Fgf‐pErk1/2 signaling enriched the nuclear translocation of Yap. Compared with control O9‐1 NC cells, no significant changes in pYap S127 levels were observed in cells treated with FGF2 or the pERK1/2 inhibitor U0126 (Figure S5A). This data suggests that the nuclear translocation of Yap is not mediated through the canonical Hippo signaling pathway.

**FIGURE 8:**
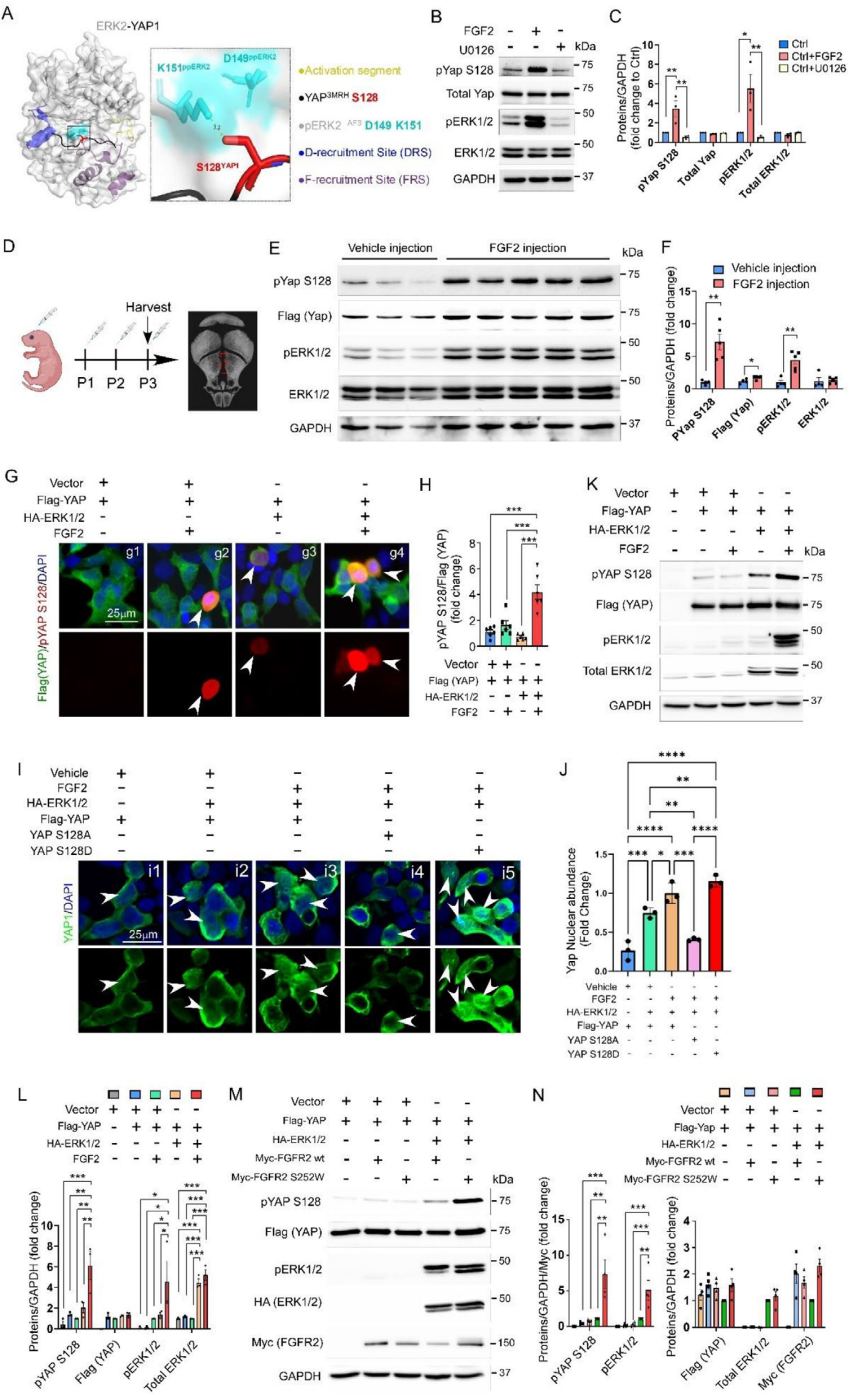
FGF signaling promotes pERK1/2 to phosphorylate Yap at S128 site. (A) ZDOCK prediction of YAPS128 with pERK2. ERK2 domain characterization adapted from Lee et al. (2011) [78]: DRS (blue) residues – 108 to 129 and 158 to 162; FRS (purple) residues – 183 to 206, 233 to 243, and 258–265; catalytic loop residues – 148 to 154 with D149 and K151 shown (cyan); activation segment (yellow) residues – 170 to 196, which overlaps slightly with the FRS. (B, C) Western blot (B) and quantification (C) of indicated proteins in O9‐1 NC cells with FGF2 or pErk1/2 inhibitor U0126 treatment. GAPDH is a loading control. *n* = 3 independent experiments. (D) Schematic shows the process of daily FGF2 injection into the frontal suture regions of mouse pups, and the red dash box indicated the frontal suture tissues we use for western blot analysis. (E, F) Western blot (E) and quantification (F) of indicated proteins in SMCs of frontal suture tissues from P3 vehicle‐ or FGF2‐injected mouse pups. GAPDH is a loading control. n = 3 vehicle‐injected pups and 5 FGF2‐injected pups. (G, H) Flag‐Tag YAP (green) and pYAP S128 (red) IF co‐staining (G), and pYAP S128 quantification (H) in 293T cells transfected with Flag‐Yap and/or HA‐ERK1/2 plasmid, following vehicle or FGF2 treatment. Cells are counterstained with DAPI (blue). White arrowheads point to the nuclear expression of pYAP S128. *n* = 3 independent experiments. Scale bar, 25 µm. (I, J) Flag‐Tag YAP (green) IF (I) and quantification (H) in 293T cells transfected with wild‐type Flag‐YAP or mutations Yap S128A or Yap S128D, and/or HA‐ERK1/2 plasmid, following vehicle or FGF2 treatment. Cells are counterstained with DAPI. n = 3 independent experiments. White arrowheads indicate nuclear expression of YAP. (K, L) Western blot (K) and quantification (L) of indicated proteins in 293T cells transfected with Flag‐YAP and/or HA‐ERK1/2 plasmid, following vehicle or FGF2 treatment. *n* = 3 independent experiments. (M, N) Western blot (M) and quantification (N) of indicated proteins in 293T cells transfected with indicated plasmids. *n* = 3 independent experiments. Data represents mean ± SEM. ANOVA combined with Tukey's multiple comparisons test was used for A, D‐E, and J. The unpaired t‐test is used to quantify C. *p < 0.05, **p < 0.01, and ***p < 0.001.

ERK1/2 belongs to the MAPK family with MAPK substrates requiring both a phospho‐acceptor (S/T)P motif, characterized by Serine (S) or Threonine (T) residues located adjacent to Proline (P) residues, and F‐site (FXFP) or D‐site (R/K)3‐5‐(X)1‐5‐Φ‐X‐Φ motif [48, 49, 50]. Importantly, we found that YAP contains ten (S/T)P motifs and four potential D‐sites (Figure S5B and C), suggesting an intriguing possibility that YAP may function as a substrate of ERK and be phosphorylated by ERK. To explore the structural basis of YAP/ERK interaction, we generated a full‐length structure of active human pERK2 using AlphaFold3 (ppERK2^AF3^). Structural quality assessment by MolProbity confirmed that our modified YAP crystal retained high structural quality (YAP: MolProbity Score = 0.50, 100th percentile). Docking with ZDOCK identified a stable complex for YAP‐pERK2 (MolProbity Score = 1.46; 96th percentile) in which YAP S128 was found to form a stable interaction, within 4Å distance, with ERK2's catalytic residues D149 and K151 (Figure 8A and Figure S5D). Computer structural modeling suggested that FGF signaling activates pERK1/2‐mediated phosphorylation of YAP at the S128 site, thereby facilitating its nuclear translocation. We then monitored the phosphorylation level of Yap at the S128 site (pYap S128) in O9‐1 NC cells after FGF2 ligand or pERK1/2 inhibitor U0126 treatment to activate or inhibit pErk1/2, respectively. Notably, we observed a dramatic increase in pErk1/2 levels and a corresponding increase in pYap S128 levels after FGF2 treatment (Figure 8B,C). When pErk1/2 was inhibited by U0126, pYap S128 levels were also repressed (Figure 8B,C). To corroborate this result in vivo, we injected FGF2 into the frontal suture region of neonatal pups of *Yap‐Tag* mice from P1 to P3 and harvested the frontal suture tissues at P3 (Figure 8D). Consistent with the robust upregulated pErk1/2 levels, total Yap and pYap S128 levels were significantly increased in the frontal suture of FGF2‐injected pups compared to vehicle‐injected controls, as indicated by western blot data (Figure 8E,F).

We further evaluated pYap S128 levels following transfection of human Flag‐YAP and/or HA‐ERK1/2 plasmids in 293T cells treated with FGF2 or vehicle (Figure 8G,H). Consistent with the findings above, pYAP S128 was localized in the nucleus of cells (indicated by white arrowheads, Figure 8G). The expression level of pYAP S128 was significantly increased in cells co‐transfected with Flag‐YAP and HA‐ERK1/2 and treated with FGF2 (Figure 8G_g4,H), compared to the group transfected with only Flag‐YAP and treated with FGF2 (Figure 8G_g2), or the group transfected with both Flag‐YAP and HA‐ERK1/2 but without FGF2 treatment (YE) (Figure 8G_g3).

To determine the importance of the Yap S128 site in Yap nuclear localization, we generated mutagenesis at the S128 site, including S128A (phospho‐deficient) and S128D (phospho‐mimetic) mutants. 293T cells transfected with HA‐ERK1/2 and YAP S128D and treated with FGF2 (Figure 8I_i5,J) exhibited the greatest increase in YAP nuclear translocation compared with other groups (Figure 8I_i1‐4,J). In contrast, cells transfected with HA‐ERK1/2 and YAP S128A displayed markedly reduced Yap nuclear translocation even with FGF2 treatment (Figure 8I_i4,J). Additionally, western blot results showed that compared to all other groups, pERK1/2 and pYAP S128 levels were significantly increased in the group with Flag‐YAP, HA‐ERK1/2, and FGF2 (Figure 8K,L). These data reveal that phosphorylation at the S128 site plays a key role in YAP nuclear translocation and that activated pERK1/2 are required for YAP phosphorylation at this site.

Next, we investigated the role of pYap S128 in NC cell‐derived osteogenesis. We transfected either an empty plasmid (vector), a WT YAP plasmid (Flag‐YAP), or the YAP S128D or YAP S128A mutated plasmids into *Yap KO* O9‐1 NC cells, to eliminate the influence of endogenous Yap on osteoblast differentiation. WT O9‐1 NC cells transfected with the vector were used as controls. These cells were cultured under osteoblast differentiation medium and were harvested at d5 of osteogenesis. We found that, similar to WT cells, *Yap KO* cells transfected with Flag‐YAP exhibited higher levels of the osteogenic marker Col1a1 (Figure S6A and B). In contrast, *Yap KO* cells expressing either YAP S128A or YAP S128D mutants showed less Col1a1 staining, comparable to the Col1a1 staining of *Yap KO* cells transfected with the vector (Figure S6A and B). These data indicated that both Yap S128A and Yap S128D mutants impair osteogenesis in NC cells, suggesting that in addition to YAP nuclear translocation, the S128 site is also important for YAP function in NC cell derived osteogenesis.

Together, these data suggest that FGF signaling promotes YAP nuclear translocation through pERK1/2‐mediated phosphorylation at the S128 site.

### 2.14 *FGFR2* S252W Mutation Facilitates ERK1/2 Phosphorylation and pYAP S128 Levels

To further assess whether the mechanism described above also applies to other activation of FGF signaling models, we examined the *FGFR2 S252W* mutation. We transfected 293T cells with Myc‐FGFR2 wt and/or Myc‐*FGFR2* S252W mutant plasmids, along with Flag‐YAP and/or HA‐ERK1/2 simultaneously (Figure 8M and Figure S6). Remarkably, we observed an increase of nuclear pYAP S128 expression of cells (white arrowheads) transfected with Myc‐*FGFR2* S252W along with Flag‐YAP and HA‐ERK1/2 plasmids, in comparison to all the other groups of cells, including those co‐transfected with Myc‐FGFR2 wt, Flag‐YAP, and HA‐ERK1/2 plasmids, as well as those with Myc‐*FGFR2* S252W and Flag‐YAP but without HA‐ERK1/2 plasmids (Figure S7). Consistently, our western blot displayed significantly increased pERK1/2 and pYAP S128 levels in cells transfected with Myc‐*FGFR2* S252W, Flag‐YAP, and HA‐ERK1/2 plasmids, compared to cells transfected with Myc‐FGFR2 wt, Flag‐YAP, and HA‐ERK1/2 plasmids, as well as all the other groups of cells (Figure 8M,N). Notably, in the absence of HA‐ERK1/2, the level of pYAP S128 was remarkably reduced regardless of transfecting with the Myc‐*FGFR2* S252W mutant or Myc‐FGFR2 wt plasmids (Figure 8M,N). These data suggest that the *FGFR2* S252W mutation also functions through pERK1/2 to phosphorylate YAP at the S128 site, thereby mediating YAP activity.

## 3 Discussion

**FIGURE 9:**
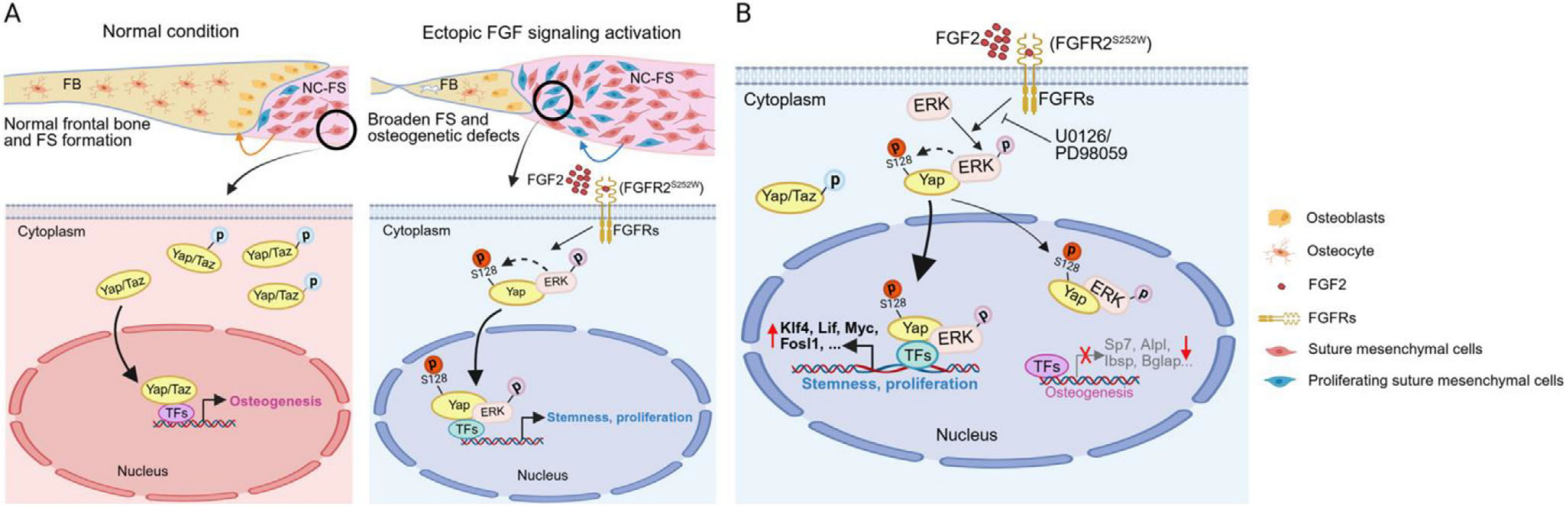
Model depicting how FGF signaling regulates stemness, proliferation, and osteogenesis of NC‐derived SMCs through Yap/Taz. (A, left) Illustration depicting how, under normal conditions, the Hippo‐signaling downstream effectors Yap/Taz regulate osteogenesis for proper frontal bone and suture formation. (A, right) Illustration depicting that ectopic FGF signaling leads to a broadened frontal suture and osteogenic defects, due to the novel regulation of Yap through the S128 site via ERK for the promotion of proliferation and stemness. (B) Illustration of the detailed mechanism identified in the ectopic FGF signaling model (A, right). In brief, ectopic FGF signaling (representative of the FGFR2^S252W^ mutation) promotes the phosphorylation of Yap at the S128 site by phosphoryated‐ERK1/2 (pERK1/2), which in turn promotes the regulation of stemness and proliferation‐related genes (Klf4, Lif, Myc, Fosl1), potentially through regulating their chromatic occupancy, over the promotion of key osteogenic genes (Sp7, Alpl, Ibsp, Bglap). This mechanism was inhibited by pERK‐inhibitors (U0126/PD98059). FB, frontal bone; NC‐FS, neural crest‐derived frontal suture; TFs, transcription factors. Illustration created using BioRender.

The FGF and Hippo signaling pathways are crucial in regulating stem cell pluripotency, self‐renewal, and differentiation, including NC cells and their lineages [33, 35]. In this study (summarized in Figure 9), our findings support a model in which FGF‐ERK signaling enhances YAP nuclear localization and chromatin engagement, promoting stemness while repressing osteogenesis in NC‐derived SMCs. We unveil that Yap/Taz, the primary downstream effectors of the canonical Hippo signaling pathway, function as noncanonical mediators of FGF signaling in NC cells and NC‐derived SMCs. We uncover a previously uncharacterized pERK1/2 phosphorylation on S128 of YAP, which promotes YAP nuclear translocation and thereby modulates YAP chromatin occupancy on key target genes, including those essential for cell cycle regulation, stemness, and differentiation.

The FGF downstream MAPK pathway (ERK1/2, p38, and JNK) contributes distinctly to the pluripotency of NC cells [14] and human induced pluripotent stem cells [13]; however, ERK1 was also observed to promote osteogenesis in human osteoblasts [51], indicating potential context‐specific roles. In the mouse sagittal suture, FGF2 also has varying effects depending on cell maturity: FGF2 promotes matrix mineralization and osteocalcin production in more mature cells while inhibiting osteoblast activity in SMCs [52]. Moreover, Erk1/2 activation has been shown to promote osteogenesis in MC3T3‐E1 cells, an osteoblast precursor cell line derived from mouse calvaria [53]. Similarly, Yap/Taz are crucial for maintaining stem cell traits in normal tissues, and Yap has been described as a “stemness” gene based on the transcriptional profiling of adult neural, hematopoietic, and embryonic stem cells [23, 54, 55]. Taz and Yap have been observed to play redundant, sometimes distinct, or even opposing roles depending on the context and time window [56]. In bone marrow‐derived mesenchymal cells, *Yap/Taz* deficiency causes osteogenic repression and adipogenesis activation in humans [56] and in mice [57]. However, *YAP1* overexpression inhibits both osteogenesis and adipogenesis in human bone marrow‐derived mesenchymal stem cells [57], highlighting the complexity of their roles. Furthermore, Yap and Taz maintained the multipotency of NC cells, yet promoted osteogenesis after NC‐derived mesenchymal cells were specified as osteochondral progenitors [33], highlighting their stage‐dependent functions. We previously found that functional redundancy of Yap and Taz in the NC and their NC‐specific deficiency resulted in bone loss in mice [33]. Our data here showed that Fgf‐induced upregulation of Yap/Taz activity also resulted in osteogenic defects. In addition, *Yap/Taz* dKD can restore the osteogenic defects caused by FGF2 treatment, yet *Yap KO* and *Taz* KD cannot. These data indicated the importance of a stage‐dependent, finely tuned level of Yap/Taz during development.

The complex signaling pathways that regulate SMCs remain a fundamental biological question. Researchers have explored the cellular changes of paraxial mesoderm‐derived coronal sutures caused by FGF signaling dysregulation [58], and different FGF signaling components exhibited distinct, and sometimes even contrasting, regulatory functions that were context‐ or stage‐dependent [36, 52, 59, 60, 61, 62], suggesting intricate molecular mechanisms underlying FGF signaling regulation in SMCs. Nevertheless, cellular and molecular alterations in the NC‐derived frontal suture following FGF signaling dysregulation are largely unknown. Our study revealed that ectopic Fgf activation in NC‐derived SMCs enhanced their stemness and proliferation while repressing osteogenesis, resulting in broadened frontal sutures and defective frontal bone formation of neonatal mice. These findings align with the frontal suture phenotype identified in Apert syndrome *Fgfr2^+/^* ^S252W^ mice. A similar midline sutural defect (open fontanelle) is also observed in infants with Apert syndrome [63], supporting the potential applicability of our study to FGFR2 gain‐of‐function mutations in both mice and humans. Importantly, SMCs are not only essential for craniofacial development but also play key roles in bone repair and regeneration. Therefore, in addition to therapeutics for birth defects, our findings could also benefit regenerative medicine.

The phosphorylation of YAP regulates its localization (nuclear vs. cytoplasmic), stability, and activity, particularly in various diseases such as cancer, fibrosis, and regenerative disorders, with abnormal YAP activation being a major driver [64]. The most common phosphorylation of YAP is at the S127 site by the canonical Hippo kinases LATS1/2. S127 phosphorylation is broadly associated with a repression of YAP activity, which promotes the cytoplasmic retention and degradation of YAP. However, our study found that pERK1/2 promoted YAP nuclear translocation by phosphorylating YAP at the S128 site. The MAPK family, including ERK1/2, phosphorylates Serine or Threonine residues located adjacent to Proline residues in its substrates [65]. Reports showed that, induced by osmotic stress, NLK, an atypical member of the MAPK kinase family [66], can also phosphorylate YAP at S128 to promote YAP nuclear translocation by blocking S127 phosphorylation and binding with 14‐3‐3 proteins [67]. Our data provided the first evidence that pERK1/2 phosphorylates YAP at S128, while not blocking the Hippo kinase‐dependent S127 site, suggesting a noncanonical mechanism by which ERK activates YAP that may operate in parallel to, or independently of, canonical Hippo signaling. This observed difference from NLK phosphorylation on YAP S128 could be due to a difference in ERK and NLK kinase activity, or a difference between cell contexts. The identification that pERK1/2 regulates YAP S128 phosphorylation represents a significant advancement in understanding YAP biology and proposes new avenues for therapeutic opportunities. Importantly, we observed elevated pYAP S128 levels in FGFR2 S252W mutant cells, implying that this mechanism is applicable in the FGFR2 S252W mutation‐associated Apert syndrome with potential impact on broader FGF signaling gain‐of‐function mutations. Thus, pERK1/2‐mediated phosphorylation of YAP S128 represents a potential druggable node to fine‐tune the function of YAP, advancing both our mechanistic understanding and therapeutic strategies.

Lastly, ERK1/2 loss‐of‐function in osteochondral progenitors was shown to block osteoblast cell fate and led to ectopic chondrogenic differentiation within the bone‐forming region of the perichondrium [68]. Although this finding was obtained in a different context, it concurs that *Yap/Taz* deficiency in NC cells leads to chondrogenesis rather than osteogenesis [33], suggesting similar roles of ERK1/2 and Yap/Taz in promoting osteogenesis. Here, our integrative analysis indicated that more than half of the differentially expressed genes in SMCs from *Fgfr2^+/^* ^S252W^ mice were potential Yap target genes, likely regulated through the coordination we identified between Erk1/2 and Yap/Taz. However, whether the interaction between Erk1/2 and Yap/Taz involves additional factors remains to be determined. Additionally, crosstalk between FGF and Hippo signaling has been reported in other contexts, including liver tumors [69]. We also validated FGF‐activated pERK‐dependent phosphorylation of YAP in several human cell types, suggesting that our findings on YAP/TAZ‐mediated FGF–ERK signaling may be applicable in other biological contexts.

In summary, our study uncovers a previously uncharacterized mechanism by which FGF signaling regulates NC‐derived mesenchymal cells through ERK‐dependent YAP nuclear translocation, modulating the balance between stemness and osteogenesis, which may occur through the phosphorylation of YAP at the S128 site. By establishing a direct molecular link between YAP regulation and the fundamental FGF signaling, these findings significantly advance the mechanistic understanding of SMC molecular regulation. Conserved FGF‐YAP regulation in human NC‐derived mesenchymal cells, HEK293 kidney cells, and HaCaT epidermal keratinocyte cells further provides valuable insights into the regulation of other stem cell types and diseases. This work opens new avenues for developing diagnostic tools and therapeutic strategies targeting NC‐related diseases and provides broader insights into stem cell regulation across various biological systems.

### 3.1 Limitations of the Study

We acknowledge several limitations in this study and recognize important opportunities for future investigation. Consistent with the premature fusion phenotype seen in the coronal sutures of FGFR2 gain‐of‐function mutations, treating the coronal sutures of E15 mouse heads with FGF2‐soaked beads also led to premature fusion, along with reduced proliferation and increased osteopontin expression in the suture mesenchyme [59]. These findings differ from those observed in our FGF2‐treated frontal sutures, reinforcing that the molecular mechanisms regulating SMC functions are suture‐specific. Moreover, recent scRNA‐seq studies have established that SMCs are a heterogeneous population composed of distinct subpopulations exhibiting cellular diversity [37]. By comparing scRNA‐seq datasets from the coronal [9] and frontal sutures [37], Farmer et al. revealed that both sutures likely contain similar cell types, yet their gene expression profiles and spatial organization are suture‐specific [9]. Such differences may lead to distinct responses of SMCs to FGF signaling in each suture, ultimately resulting in divergent phenotypes, highlighting the need for further in‐depth investigation.

We observed that FGF‐activated pERK1/2 can promote YAP nuclear transcriptional activity to regulate genes involved in stemness, proliferation, and osteogenesis. Whether FGF‐induced suppression of osteogenesis reflects impaired differentiation or sustained stemness remains unclear. Furthermore, the transcription factor networks and transcriptional contexts underlying the ERK‐YAP interaction likely incorporate additional signaling pathways and transcription factors and may further differ across cell lineages due to lineage‐specific regulatory mechanisms. Therefore, a hierarchical model in which ERK‐YAP signaling maintains NC cells in a progenitor state, thereby preventing osteogenic specification, warrants future investigation. The developing craniofacial structures undergo dynamic changes and establish distinct biomechanical environments, regulated by multiple signaling pathways including Hippo, FGF, BMP, Notch, and Wnt, as well as mechanical cues. Disruption of this finely tuned process can lead to pathological conditions such as craniosynostosis. There is established crosstalk between Hippo and other signaling pathways, such as that Yap/Taz can interact with the Wnt signaling downstream effector β‐catenin to regulate gene transcription in the NC cells [33]. Although our findings indicate a direct pERK1/2–YAP interaction, it is worthwhile to investigate whether other scaffold proteins facilitate this interaction. It is also worth investigating if the pERK1/2‐YAP regulated transcriptional activity involves other known and unknown factors, such as the NC‐specific transcription factors TFAP2A and PAX3. In addition to biochemical signals, cranial sutures particularly experience suture‐specific mechanical forces such as tension from brain growth, which influences suture cell fate, driving osteogenic versus patency programs and the timing of fusion [70]. Since YAP is a key mechanotransducer that senses and responds to mechanical stimuli, and cranial sutures are known to have biomechanical sensitivity, it will be important to assess whether ERK‐mediated YAP activation intersects with mechanotransductive inputs such as FAK‐integrin signaling, ECM stiffness, or cytoskeletal dynamics, in NC‐derived SMCs during cranial suture formation. Furthermore, it will be valuable to integrate our bulk RNA‐seq and CUT&Tag‐seq datasets with recently published transcriptomic data of NC and suture lineages to further elucidate the relationship between Yap chromatin binding and other signals, including NC‐specific transcription factor networks and mechanical signals. In addition, noncoding cis‐regulatory element dynamics play an essential role in transcriptional landscapes [71], which may be associated with the FGF‐induced YAP target gene transcription and functional changes. Additional studies, such as single‐cell multi‐omics and integrated analysis, will be needed to gain deeper insight into how FGF signaling regulates diverse functions through YAP and ERK1/2, and other potential mechanisms. It is also worth performing ChIP‐seq or CUT&RUN‐seq experiments to evaluate whether ERK inhibition affects YAP chromatin association to gain more mechanistic insights into how ERK activity modulates the transcriptional function of YAP.

In addition, there are limitations and potential artifacts arising from our in vitro studies, and further investigation will be valuable to better understand the FGF‐YAP axis. Transfection assays in 293T and HaCaT cells revealed conserved FGF‐YAP regulation, yet have possible overinterpretation of exogenous systems; therefore, further in vivo validations for the in vitro findings are still needed. We also observed that the *FGFR2 S252W* gain‐of‐function mutation can facilitate ERK1/2 phosphorylation of pYAP S128 levels, and *FGFR2 S252W* mutant cells had elevated pYAP S128 levels. This promising in vitro finding implies an applicable mechanism in Apert syndrome and potentially other FGF gain‐of‐function associated diseases. In future works, we will obtain mouse models with FGF gain‐of‐function mutations, such as the *Fgfr2^+/^* ^S252W^ mouse model, and further study the Yap/Taz‐mediated Fgf‐Erk signaling in vivo. In addition, to strengthen our conclusions regarding therapeutic potential, it would be worthwhile to perform longitudinal studies on mice treated with pERK inhibitors to evaluate the long‐term developmental impact. Therapeutic modulation of the ERK‐YAP axis may hold promise for craniosynostosis or other FGF‐hyperactivation disorders; however, strategies will require careful consideration of tissue specificity, developmental timing, and potential consequences for stem cell maintenance. Additionally, we showed that disturbed Yap S128 site phosphorylation impaired NC cell‐derived osteogenesis in vitro, but we did not investigate its role in other downstream biological functions of the FGF‐YAP axis. In future studies, it will be valuable to investigate the impact of YAP S128 in biological functions, such as proliferation and stemness of NC and NC‐derived lineages, and the potential differences, as well as whether phosphorylation of S128 influences the phosphorylation of S127, 14‐3‐3 binding, or LATS kinase activity, and the potential variations in different cell contexts. Further studies regarding the function and regulation of Yap S128 in vivo will also be valuable.

## 4 Materials And Methods

### 4.1 Transgenic Mouse Lines and Alleles

The Wnt1‐Cre, *Yap^flox/flox^*, and *Taz^flox/flox^* mouse lines and alleles have been described previously [72]. The mTmG reporter mice (Stock JAX: 007676) were from Jackson Laboratories. *Yap^Tag/Tag^* mouse line was generously provided by Dr. James Martin's lab. All mice were housed in the animal facility of the University of Texas Health Science Center at Houston. The experimental protocol was reviewed and approved by the Animal Welfare Committee (AWC) and the Institutional Animal Care and Use Committee of the University of Texas Health Science Center at Houston (AWC‐22‐0049).

### 4.2 FGF2 and U0126 Inhibitor Injection

The FGF2 protein (R&D Systems, 233‐FB‐025) was reconstituted at 25 µg/mL in sterile PBS containing 0.1% bovine serum albumin (BSA). The U0126 (Cell Signaling Technology, 9903) was reconstituted at 50 mm in DMSO. Subcutaneous injections into the frontal suture region of neonatal mouse pups were performed each day from postnatal day 1 (P1) to P3 or P7. Subcutaneous injection of the vehicle (sterile PBS containing 0.1% BSA or DMSO) was used as the control. The head samples from P3 and P7 pups after injection were harvested for further experiments. The dosage was 10 uL per pups for both FGF2 and U0126 injection.

### 4.3 Micro‐CT Analysis

The hair and skin were removed from the mouse head samples, which were then washed with phosphate‐buffered saline (PBS). The head samples were fixed overnight at 4°C using 4% paraformaldehyde (PFA) followed by 70% ethanol. The samples were scanned using a Bruker Skyscan 1276 scanner. The cranial skull structure, frontal bone mineralization, and frontal suture width were measured using the CT Analyzer software.

### 4.4 Histological Analysis and Immunofluorescence Staining

All mouse heads were dissected in PBS and fixed in 4% PFA overnight at 4°C. The heads were decalcified in Cal‐Ex Decalcifier solution (Thermo Fisher Scientific; CS5101D) and then were processed and sectioned into 7 µm thick paraffin sections. The slides were deparaffinized and hydrated in gradient ethanol (100%, 95%, 70%) and distilled water. Then, Hematoxylin and eosin (H&E) staining and immunofluorescence staining (IF) of these sections were performed. Primary antibodies used for IF: Myc‐Tag (9B11) (Cell Signaling Technology, 2276,1:300), Yap (Santa Cruz Biotechnology, sc‐10199, 1:150), Anti‐Phospho‐p44/42 MAPK (Erk1/2) (Cell Signaling Technology, 9101, 1:200). Slides were visualized by using a Zeiss LSM Confocal Microscope.

### 4.5 O9‐1 NC Cell Culture and Manipulation

O9‐1 NC cells were obtained from Robert E. Maxson's lab, which was a primary cell line isolated from the head of E8.5 mouse embryos [38]. The rationale for using O9‐1 NC cells in this study is to mimic NC cells in vitro, due to their multipotent capacity to differentiate into multiple cranial neural crest derivatives, including osteoblasts, chondrocytes, smooth muscle cells, and glia [38]. All O9‐1 NC cells for experiments were contamination‐free. O9‐1 NC cells were cultured under a nondifferentiation condition based on a previously described protocol [38]. The osteoblast differentiation conditions used for O9‐1 NC osteogenesis were also described previously [38]. For the knockdown (KD) experiments mediated by siRNA, cells were transfected with *Yap* (Dharmacon, L‐046247‐01‐0005) and/or *Taz* (Dharmacon, L‐041057‐01)‐targeted siRNA SMART pools. Scramble siRNA (Dharmacon, D‐001810‐04‐05) was used as a control. KD experiments were performed according to the guidelines of the RNAiMAX transfection procedure (Life Technologies, 13778075). For *Yap* KO O9‐1 NC cells, exon 3 of *Yap* was removed using CRISPR/Cas9 genome editing, as detailed in a previous report [73]. Mycoplasma contaminants were evaluated in the cell line.

### 4.6 Ex Vivo Cell Culture and Differentiation

Frontal suture mesenchymal cells were dissociated from the frontal suture regions of E13.5 embryos. Briefly, frontal suture tissues were finely minced by using dissecting scissors and then digested in HBSS containing 0.25% dextrose (Sigma‐Aldrich, G8769) and 0.5% collagenase (Sigma‐Aldrich, 10269638001) at 37°C for 1 h. The cells were dissociated through gentle pipetting, centrifuged, and then resuspended into BGJb medium with 10% fetal bovine serum (FBS) and 1% penicillin‐streptomycin. The cells were cultured on 0.05% Matrigel‐covered plates and incubated in a standard cell culture incubator (37°C, 5% CO_2_, 5% O_2_). Following 48 h of culture, the cells were detached with 0.25% trypsin, centrifuged, and then seeded into 24‐well plates at a density of 15 000 cells/cm^2^. On the following day, osteoblast differentiation was triggered by switching the medium to osteoblast differentiation medium, as previously described [38]. These cells were induced for 10 days, and the osteoblast differentiation medium was replaced every other day. All cells were contamination‐free.

### 4.7 Human Embryonic Stem Cell (hESC) Culture and Maintenance

Human embryonic stem cell line WA09 (H9) was cultured in mTeSR1 medium (Stem Cell Technologies) on Matrigel (Corning) coated plates and incubated in a standard cell culture incubator (37°C, 5% CO_2_, 5% O_2_). Cells were passaged at 80% confluency with Accutase (Stem Cell Technologies). All cells were contamination‐free.

### 4.8 Cranial NC Differentiation of hESCs

The differentiation method followed the protocol from Leung et al. [74], with minor variation. hESCs cultured to 80%–90% confluency were washed with 1X PBS and dissociated in Accutase. Cells were quenched with warmed NC differentiation media (1X B27 (2%), 1X Glutamax, and 0.5% BSA in DMEM/F12) with 10 µm Y‐27632 (TORCIS, 1254) and centrifuged at 1200 RPM for 4 min at room temperature. The cell pellet was resuspended to a single cell suspension using NC differentiation media with 10 µm Y‐27632. Cells were plated on pre‐coated Matrigel culture plates at a density of 4×10^4^ cells/cm^2^ per culture plate, supplemented with CHIR99021 (TORCIS, 4423) to a final concentration of 3 µm. Media was changed daily. Media on Day 1 (1 day after plating) was supplemented with 10 µM Y‐27632 and CHIR99021 to a final concentration of 3 µm. Days 2–5, media was replenished with NC differentiation media. By Day 5, cells were ready to be used for analysis or undergo further differentiation. All cells for experiments were contamination‐free.

### 4.9 Osteogenic Differentiation of hESC‐derived Cranial NC Cells and Manipulation

Differentiation protocol followed the method described by Gomez et al. [75]. In brief, cells were plated in a single cell suspension at a density of 3×10^5^ cells/cm^2^ in osteogenic differentiation media (α‐MEM containing 10% FBS, 10 mm β‐glycerol phosphate, 0.1 µm dexamethasone, 200 µm Ascorbic Acid), supplemented with 10 µm Y‐27632. Osteogenic differentiation media were changed every 3 days for 21 days. For the KD experiments mediated by siRNA, cells were transfected with Scramble siRNA (Dharmacon, D‐001810‐04‐05) as a control or *Erk1* (Mapk3, Dharmacon, L‐040613‐00‐0005) and *Erk2* (Mapk1, Dharmacon, L‐040126‐00‐0005)‐targeted siRNA SMART pools. All KD experiments were performed according to the guidelines of the RNAiMAX transfection procedure (Life Technologies, 13778075). For treatment experiments, the FGF2 protein (R&D Systems, 233‐FB‐025) was reconstituted at 25 µg/mL in sterile PBS containing 0.1% bovine serum albumin (BSA), and the vehicle consisted of sterile PBS containing 0.1% BSA). All cells used in experiments were contamination‐free.

### 4.10 HaCaT Cell Culture and Manipulation

HaCaT cells (human epidermal cells) were cultured in media consisting of DMEM (Corning), 10% FBS, 2 mm L‐Glutamine, 1.0 mM sodium pyruvate, and pen/strep, and cultured in a standard cell culture incubator (37°C, 5% CO_2_/5% O_2_). Cells were passaged at 80% confluency with 0.25% Trypsin EDTA (Corning, 25‐053‐CI). KD experiments followed the guidelines of the RNAiMAX transfection procedure (Life Technologies, 13778075) and were mediated by either Scramble siRNA (Dharmacon, D‐001810‐04‐05) as a control or *Erk1* (Mapk3, Dharmacon, L‐040613‐00‐0005) and *Erk2* (Mapk1, Dharmacon, L‐040126‐00‐0005)‐targeted siRNA SMART pools. Treatment experiments utilized the FGF2 protein (R&D Systems, 233‐FB‐025) reconstituted at 25 µg/mL in sterile PBS containing 0.1% bovine serum albumin (BSA). All cells were contamination‐free.

### 4.11 Von Kossa Staining

Twenty one days after osteoblast differentiation, the H9 hESC‐derived NC cells were washed twice with PBS, fixed in 4% PFA for 10 min at room temperature, and washed three times with PBS. Next, the cells were stained using a Von Kossa staining kit (Abcam, ab150687) according to the manufacturer's guidelines. Briefly, the cells were washed with distilled water once and then incubated in 5% silver nitrate solution for 60 min with exposure to ultraviolet light. Following washing 2 times with distilled water, the cells were incubated in 5% sodium thiosulfate for 5 min and were then rinsed with water. The cells were next counterstained with nuclear fast red (Sigma Aldrich, N8002‐5G) for 5 min. After 2 min of tap water rinsing and 2 times of distilled water washing, the cells were kept in distilled water. Images were acquired using the LAS X imaging system (Leica).

### 4.12 Coimmunoprecipitation and Western Blotting

To detect the interaction between Yap and p‐ERK1/2, we conducted coimmunoprecipitation (Co‐IP) experiments with O9‐1 NC cells and 293T cells. Cells were harvested when the confluency reached 90%. For O9‐1 NC cells, the nuclear proteins were extracted using EpiQuik Nuclear Extraction Kit (EpiGentek, OP‐0002‐1) according to the manufacturer's guidelines. The nuclear extracts were then incubated with anti‐Yap antibody (Novus, NB110‐58358) or anti‐rabbit‐IgG antibody (Millipore Sigma, 12370) overnight at 4°C. For 293T cells, 0.5% NP‐40 lysis buffer (50 mm Tris‐HCl [pH 7.5], 150 mm NaCl, 0.5% NP‐40, 10% glycerol, and phosphatase and protease inhibitors) was used to lyse cells. And then, the whole cell proteins of 293T cells were extracted and incubated with anti‐Flag antibodies (Abcam, ab1162) overnight at 4°C. Next, Protein G Dynabeads (Invitrogen, 10003D) were added to the extracts of O9‐1 NC cells and 293T cells. The immunocomplexes with different antibodies were washed 4 times with cold 0.5% NP‐40 lysis buffer and collected in 20 µL of 2X Laemmli sample buffer (Bio‐Rad, 1610747) supplemented with 2‐mercaptoethanol (Bio‐Rad, 1610710). For the following, the western blot analysis has been described previously [33].

Primary antibodies used in western blotting were anti‐Phospho‐Yap1‐S128 antibody (Abclonal, AP1187, 1:1000), anti‐Yap/Taz antibody (Cell Signaling Technology, 8418, 1:1000), anti‐Phospho‐p44/42 MAPK (Erk1/2) (Cell Signaling Technology, 9101, 1:1000), anti‐ Erk1/2 (Cell Signaling Technology, 4695, 1:1000), anti‐HDAC1 antibody (Abcam, ab53091, 1:1000), anti‐GAPDH antibody (Abcam, ab9485, 1:5000), anti‐Flag‐tag antibody (Abcam, ab1162), anti‐HA‐tag antibody (Santa Cruz Biotechnology, sc‐7392, 1:2000), and anti‐Myc‐tag antibody (Cell Signaling Technology, 2276, 1:1000).

### 4.13 In Vitro Phosphorylation Assay

In vitro phosphorylation was performed by incubating 200 ng of recombinant YAP1 (cat# H00010413‐P01, Novus) with 20 ng of recombinant, activated ERK1 (cat# 1879‐KS‐010, R&D Systems) in 1× kinase buffer containing 40 mm Tris–HCl (pH 7.4), 20 mm MgCl_2_, 0.1 mg/ml BSA, and 0.05 mm DTT. ATP (final concentration 1 µm) was added to initiate the reaction, which was conducted at room temperature for 40 min. To terminate the reaction, 2.5 µL of 0.5 m EDTA was added. The reaction products were then mixed with 22.5 µl of 2× Laemmli buffer and subjected to western blot analysis using the indicated antibodies (see Figure 3 and its Legend). A negative control reaction was prepared by adding 2.5 µL of 0.5 m EDTA to the mixture before ATP addition to quench kinase activity.

### 4.14 Mutagenesis and Cell Transfection

To perform mutagenesis of the YapS128 site for S128A (phospho‐deficient) and S128D (phospho‐mimetic), we utilized the Q5 Site‐Directed Mutagenesis Kit (New England Biolabs, E0554S), following the product protocol. Transfection was conducted 8 h after the passage of 293T cells and O9‐1 cells. Both O9‐1 cells and 293T cells were transfected with different plasmids according to the guidelines for the Lipofectamine 3000 Transfection procedure (Invitrogen, L3000015). After 48 h of incubation, 293T cells were either fixed for immunofluorescence or harvested for western blot analysis. Transfected O9‐1 cells were incubated overnight under osteogenic differentiation for 5 days, then fixed for immunofluorescence.

### 4.15 CUT&Tag Sequencing

The Cleavage Under Targets and Tagmentation (CUT&Tag) protocol was modified from the published protocol [76] and has been described previously [33]. The primary antibodies used for the CUT&Tag list below: Yap1 (1:50; Novus, NB110‐58358), H3K4me3 (1:50; Cell Signaling Technology, 9733), and anti‐rabbit IgG (1:50; MilliporeSigma, 12370). The CUT&Tag‐seq datasets have been deposited to the National Center for Biotechnology Information Gene Expression Omnibus under accession number GSE287587.

### 4.16 Sequencing Data Processing and Analysis

For CUT&Tag‐seq data processing, FASTQ format raw reads were separately aligned to the mm10 using Bowtie2 with the parameters “–local –very‐sensitive‐local –no‐unal –no‐mixed –no‐discordant –phred33 ‐I 10 ‐X 700”. Bigwig files were generated for visualization in the UCSC Genome Browser. The alignment files were then used to call peaks with SEACR, applying the “norm stringent” Settings. The stringent peak files were then annotated using the R package “ChIPseeker”, and the peak compositions were calculated to create a pie chart. The R package “Gennomation” was employed to generate a signal heatmap, with YAP peaks scaled to the 2‐kb region around the peak summit for heatmap profiling. Additionally, Gene Ontology (GO) analysis was conducted via Metascape, which provided multiple defined terms representing gene characteristics, including cellular components, molecular functions, and biological processes. Gene set enrichment analysis (GSEA) was carried out using the FGSEA R package [77] with default parameters. Proliferation and osteogenesis‐related genes were obtained from the TRUST database and Gene Ontology analysis.

### 4.17 Proximity ligation assay (PLA)

PLA was performed using the Duolink In Situ Red Starter Kit according to the manufacturer's guidelines (Sigma‐Aldrich, DUO92101). Briefly, the cells on plates were washed using PBS and fixed with 4% PFA for 10 min at room temperature. The paraffin tissue sections were deparaffinized and hydrated in gradient ethanol (100%, 95%, 70%) and distilled water. Then, the cells/tissue sections were permeabilized in PBS/Triton 0.5% for 15 min at room temperature and blocked in Duolink blocking solution for 1 h at 37°C. Next, the cells/tissue sections were incubated with primary antibodies Yap (1:100, Santa Cruz; sc‐101199) and p‐ERK1/2 (1:200, Cell Signaling Technology; 4376) overnight at 4°C. Subsequently, the cells/tissue sections were incubated with the PLA probes (60 min), ligase (30 min), and polymerase (100 min) at 37°C, separately. After washing the cells/tissue sections, they were mounted using the Duolink In Situ Mounting Medium with DAPI. Red dot fluorescence indicated the interaction of Yap and p‐ERK1/2 in cells. Results were visualized via a LAS X imaging system (Leica).

### 4.18 Bioinformatic Prediction

Human YAP1 crystal (PDB: 3MHR) and a full‐length AlphaFold2 model of ERK2 were docked using ZDOCK and HDOCK. The protein‐ligand interface was visualized using LigPlot. All models were rendered using PyMOL Molecular Graphics Software. We generated a full‐length structure of active human pERK2 using AlphaFold3 (ppERK2AF3). The predicted structure was assessed to have high quality (MolProbity Score = 0.87; 100th percentile) and shared a similar fold (TM‐score = 0.566) with the crystal structure of rat pERK2 (PDB: 2ERK), supporting the efficacy of our modeling approach. Given that full‐length YAP is extensively disordered, which complicates accurate docking analyses, we used the currently available 10 amino acid crystal peptides of YAP‐p127 (PDB: 3MRH). The structures were modified by replacing the phosphorylated serine residues (pS127 in YAP) with their unphosphorylated serine, resulting in the active form of YAP for downstream docking.

### 4.19 Quantification and Statistical Analysis

Image J software was utilized for cell counting, intensity analysis, and colocalization of immunofluorescence images, as well as for grey intensity analysis of western blot data. For immunofluorescence data, the ratio of Ki67^+^ cells was analyzed using Ki67^+^ cell number divided by the total cell number (DAPI); the mean intensity of PLA, Col1a1, Runx2, Sox2, Flag‐Yap and pYap S128 signaling was analyzed in different groups and then expressed as fold change relative to their control, respectively. Furthermore, colocalization percentages were obtained utilizing threshold‐based Manders' Coefficients from the Image J plugin JACoP. For the comparisons of two groups, two‐sample *t* tests were employed for the data that followed a normal distribution, and the Mann‐Whitney test was applied to data that did not, as noted in the figure legends. For comparisons involving more than two groups, one‐way ANOVA followed by post‐hoc Tukey's test was used for data that conformed to a normal distribution, while the Kruskal‐Wallis test with *post‐hoc* Dunn's test was applied to non‐normally distributed data, as noted in the figure legends. Western blot data were normalized to their respective controls and analyzed using one‐sample *t* tests. Mann‐Whitney tests were used for qRT‐PCR data analyzed. All statistical tests were two‐tailed, and normality assumptions were checked with Shapiro‐Wilks tests. *p*‐value (*p*) ≤ 0.05 was deemed statistically significant. In the figures, *p < 0.05, **p < 0.01, and *** *p *< 0.001. Quantification data were expressed as the mean ± standard error of the mean (SEM). All bar graphs and statistical analyses were generated using GraphPad Prism version 8.0.

## Author Contributions

X.Z., S.E., and J.W. designed the research study; X.Z., S.E., K.S, W.C., E.L., C.T., and Y. L. performed the research; X.Z., L.T., S.E., X.C., S.M.F., Z.C., E.L., K.S., M.Z., Y. L. analyzed the data; X.Z., S.E., E.L., Y. L., J.W., M.L., S.Z., J.F.M., and J.W. provided reagents and revised the manuscript; X.Z., L.T., S.E., S.M.F., and J.W., wrote the manuscript.

## Funding

The authors thank the funding sources from the National Institutes of Health (NIH) K01DE026561 (Jun W.), R01DE029014 (Jun W.), R01HL142704 (Jun W.), K99DE033506 (X.Z.), and F31HL176166 (S.E.); the Lawrence Research Award from the Rolanette and Berdon Lawrence Bone Disease Program of Texas (X.Z.). The authors thank the technical support from the Cancer Prevention and Research Institute of Texas (CPRIT RP180734). MicroCT imaging was supported by NIH S10OD030336 and performed through the MicroCT Imaging Facility at the McGovern Medical School at UTHealth.

## Conflicts of Interest

J.F.M. is a co‐founder of Medley Therapeutics (formerly Yap Therapeutics). Other authors declare no competing or financial interests.

## Supporting information




**Supporting File**: advs75207‐sup‐0001‐SuppMat.docx.
Please note: The publisher is not responsible for the content or functionality of any supporting information supplied by the authors. Any queries (other than missing content) should be directed to the corresponding author for the article.
[Correction added on 1 May 2026 after first online publication: Supplementary file has been added.]

## Data Availability

Further information and requests for resources and reagents should be directed to and will be fulfilled by the Lead Contacts, Xiaolei Zhao, (Xiaolei.Zhao@uth.tmc.edu) and Jun Wang (Jun.Wang@uth.tmc.edu). The CUT&Tag datasets for YAP1, H3K4me3 and IgG have been deposited to the National Center for Biotechnology Information (NCBI) Gene Expression Omnibus under the accession number GSE287587. Bulk RNA‐seq datasets of O9‐1 NC cells treated under various conditions at d2.5 and d5 of osteoblast differentiation have been deposited to the NCBI Gene Expression Omnibus under accession number GSE287588. The CUT&RUN datasets for YAP1, H3K27me3 and IgG have been deposited to the NCBI Gene Expression Omnibus under the accession number GSE154332. Bulk RNA‐seq datasets for O9‐1 NC cells under different conditions have been deposited to the NCBI Gene Expression Omnibus under accession number GSE287588. All other data needed to evaluate the conclusions in the paper are present in the paper and/or the Supplementary Materials.
