# Supplementary material for: ERK‐Mediated Phosphorylation of YAP Defines a Noncanonical FGF Signaling Mechanism in Stem Cells
Source: Adv Sci (Weinh). 2026 Apr 10:e11484. Online ahead of print. doi: 10.1002/advs.202511484 (PMC13334658; doi:10.1002/advs.202511484)
Supplement: Supplementary file 1 — Supporting File: advs75207‐sup‐0001‐SuppMat.docx. Please note: The publisher is not responsible for the content or functionality of any supporting information supplied by the authors. Any queries (other than missing content) should be directed to the corresponding author for the article. [Correction added on 1 May 2026 after first online publication: Supplementary file has been added.] [file ADVS-9999-e11484-s001.docx]

**SUPPLEMENTAL FIGURE AND FIGURE LEGENDS**


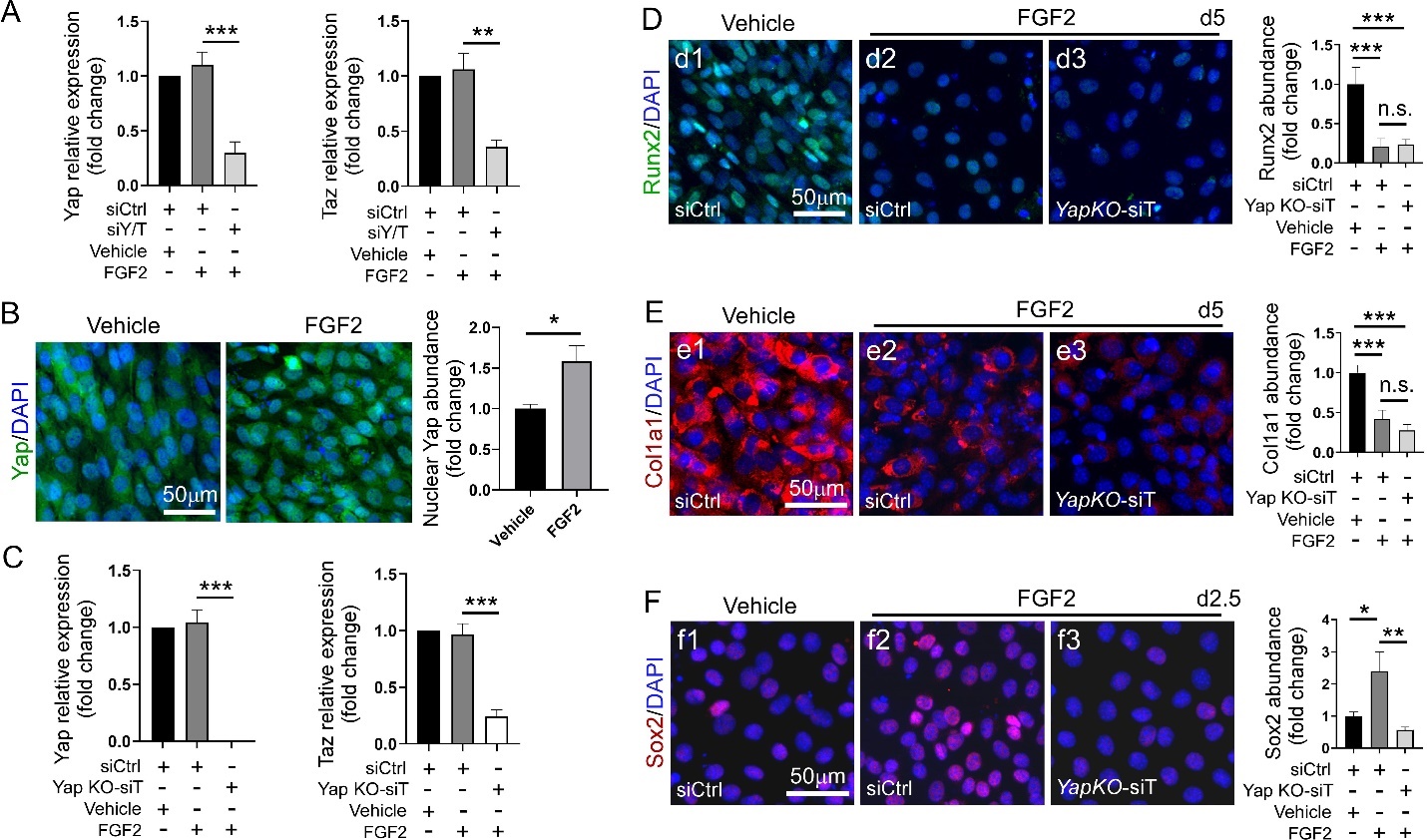


**Figure S1. The Osteogenesis and Stemness of NC-derived Mesenchymal Cells Are Yap/Taz Dosage-dependent**. (**A**) Quantification of Yap (left) and Taz (right) transcripts relative to GAPDH in siCtrl and siY/T O9-1 NC cells, with vehicle or FGF2 treatment, measured by qRT-PCR. n = 3 independent experiments. (**B**) Immunofluorescence (IF) staining for Yap (green) expression and quantification in d2.5 osteogenic induced O9-1 NC-derived mesenchymal cells, following vehicle (left) or FGF2 (right) treatment. Cells are counterstained with DAPI (blue). n = 3 independent experiments. (**C**) Quantification of Yap (left) and Taz (right) transcripts relative to GAPDH in siCtrl and *Yap KO* with Taz KD (*YapKO*-siT) O9-1 NC cells with vehicle or FGF2 treatment measured by qRT-PCR. n = 3 independent experiments. (**D**-**F**) IF staining and quantification for Runx2 (D), Col1a1 (E) and Sox2 (F) expression in siCtrl and *YapKO*-siT O9-1 NC cells at d5 (D and E) or d2.5 (F) osteogenic induction, following vehicle (d1, e1 and f1) or FGF2 (d2, d3, e2, e3, f2 and f3) treatment. Cells are counterstained with DAPI (blue). n = 3 independent experiments. Data represents mean ± SEM. ANOVA combined with Tukey’s multiple comparisons test was used for A, C, and D-F. Unpaired t-test is used to quantify B. *p < 0.05, **p < 0.01, and ***p < 0.001.


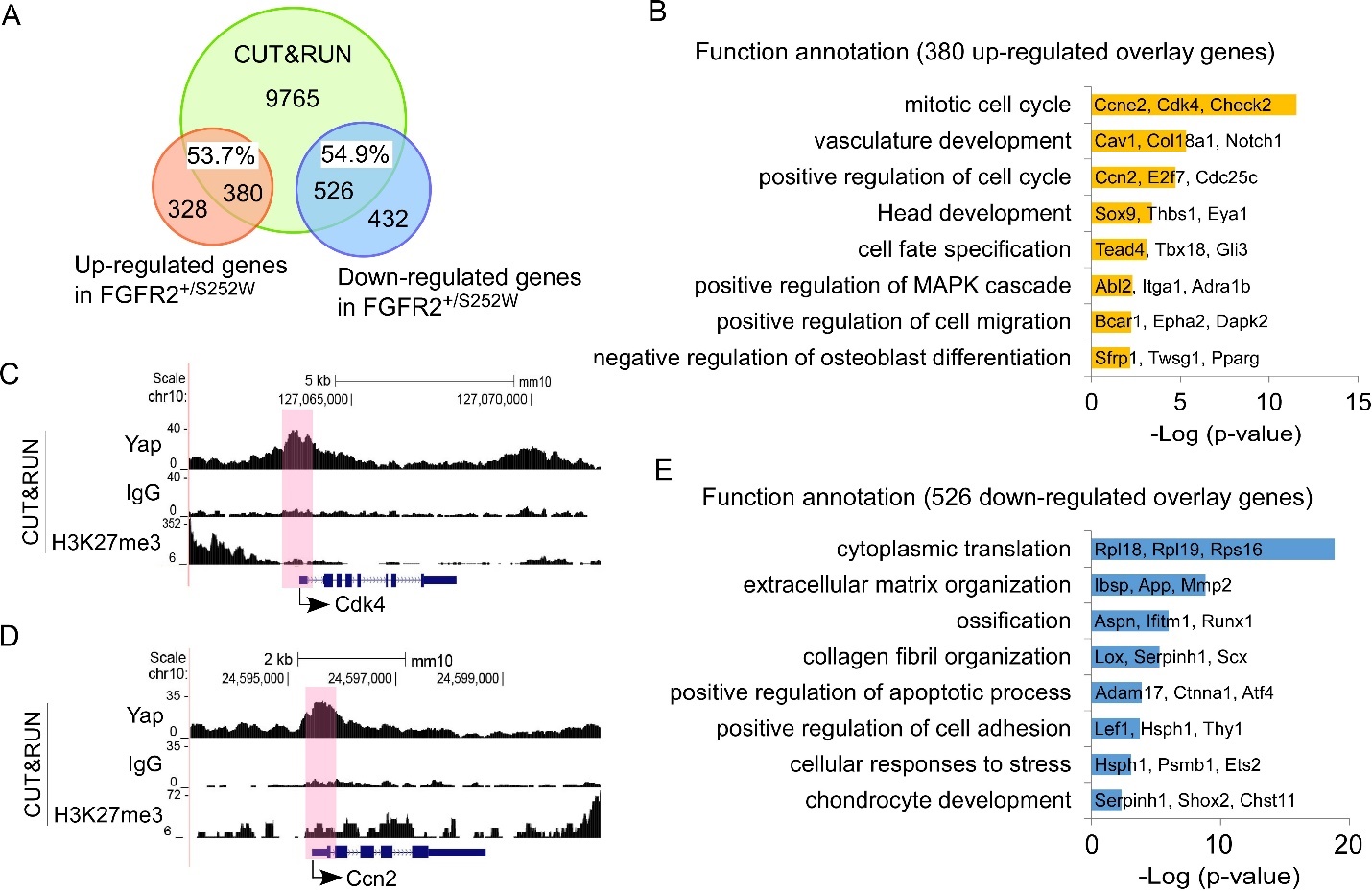


**Figure S2. Over half of the DEGs caused by** *Fgfr2^+/S252W^* **mutation in frontal SMCs and osteogenic fronts of mice are potential Yap targets**. (**A**) Venn diagram showing the overlap of Yap-bound genes identified from our published CUT&RUN-seq data (*36*) with differentially expressed genes (DEGs) in published bulk RNA-seq data from E18.5 frontal SMCs of *FGFR2^+/S252W^* mutants compared to control embryos (*41*). (**B**) Gene Ontology (GO) term analysis for overlapping genes of upregulated genes in SMCs of *FGFR2^+/S252W^* mutants. (**C**-**D**) Peak calling at overlapping genes Cdk4 (D) and Ccn2 (E) from CUT&RUN-seq. The open chromatin of the promoter regions of these genes is highlighted in pink. (**E**) GO term analysis for overlapping genes of downregulated genes in SMCs of *FGFR2^+/S252W^* mutants.


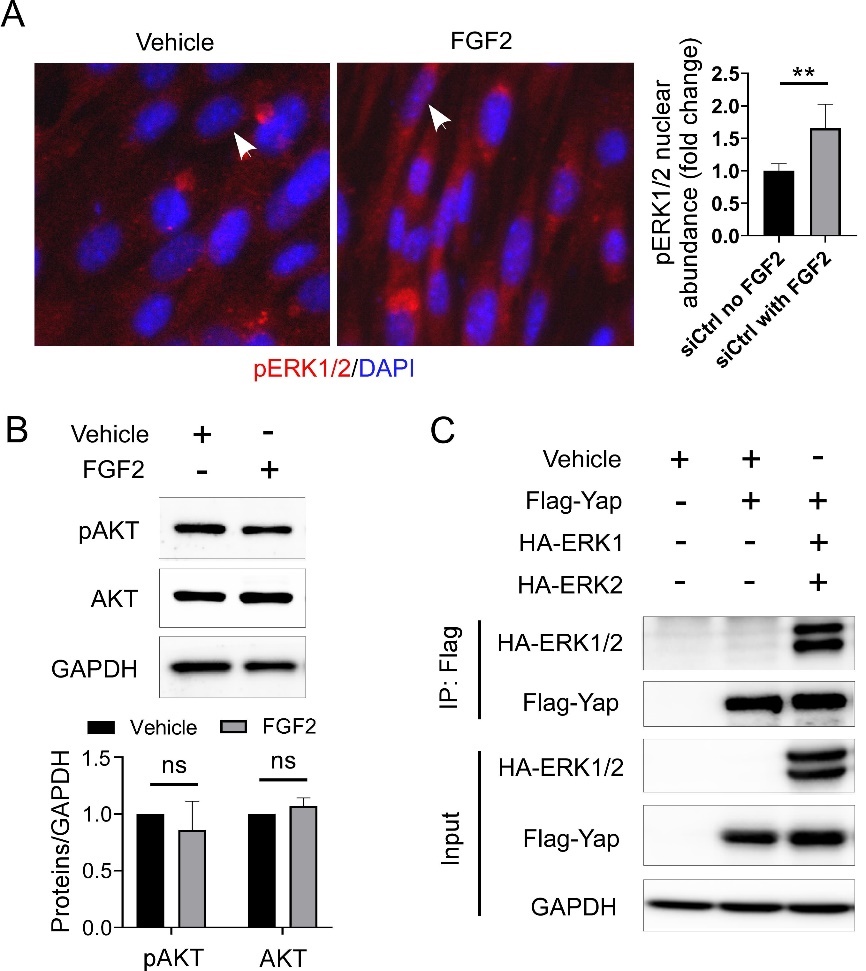


**Figure S3. Yap and ERK1/2 Interaction**. (**A**) pERK1/2 (red) expression and quantification in d2.5 osteogenic induced O9-1 NC cells, following vehicle (left) or FGF2 (right) treatment. Cells are counterstained with DAPI (blue). n = 3 independent experiments. (**B**) Western blot and quantification for pAKT and total AKT expression in d2.5 osteogenic induced O9-1 NC cells, following vehicle or FGF2 treatment. n = 3 independent experiments. (**C**) Co-immunoprecipitation (Co-IP) for HA-ERK1/2 and Flag-Yap in Flag-Yap immunoprecipitants from 293T cells transfected with Flag-Yap and/or HA-ERK1/2 plasmids. n = 3 independent experiments. Data represents mean ± SEM. The unpaired t-test is used for quantification. **p < 0.01.


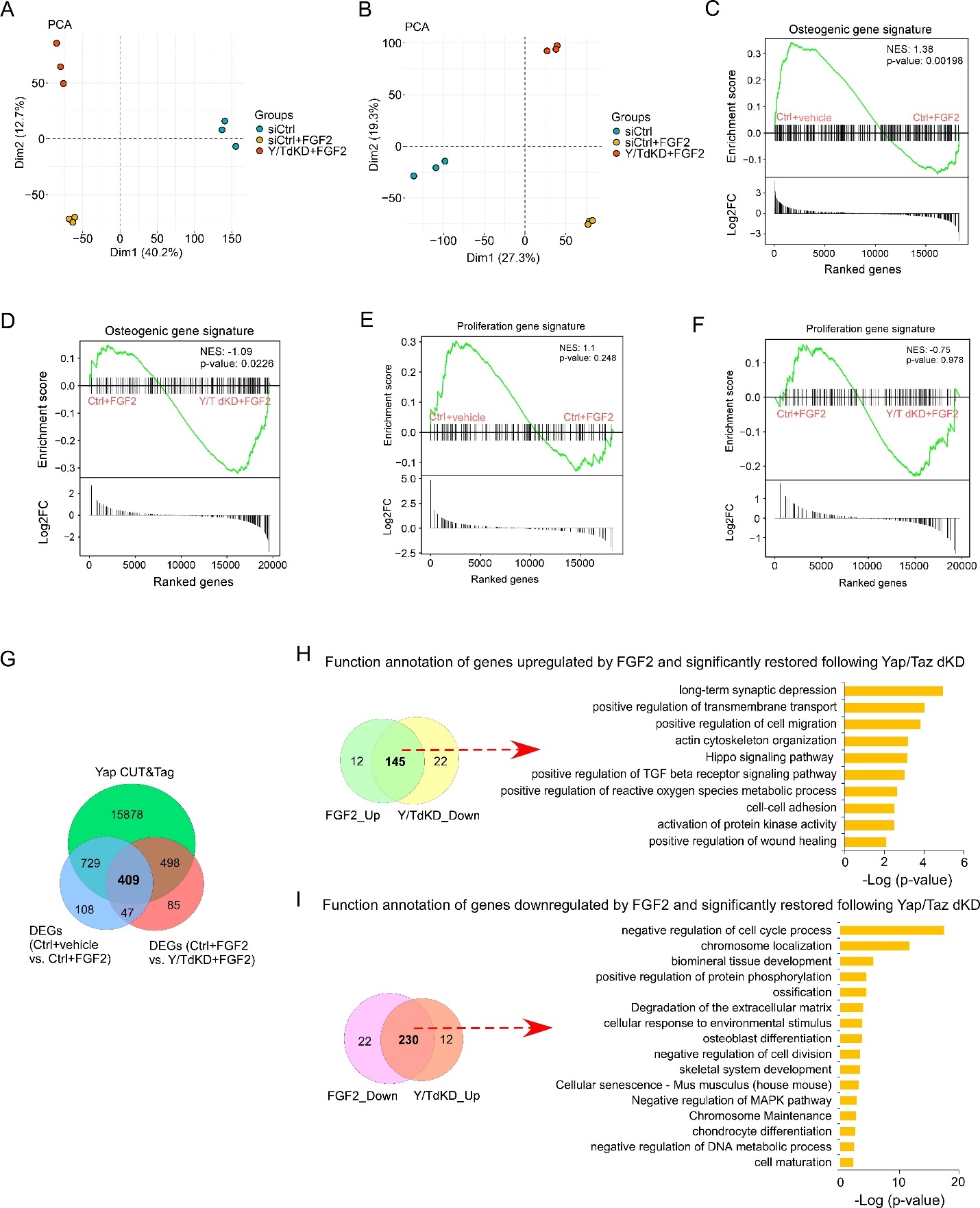


**Figure S4. FGF Signaling Regulates Stemness and Osteogenic Genes of NC-derived Mesenchymal Cells via Yap/Taz**. (**A**-**B**) Principal Component Analysis (PCA) for RNA-seq data from d2.5 (A) and d5 (B) osteogenic induced O9-1 NC cells, following different siRNA and vehicle or FGF2 treatments. (**C**-**F**) Gene Set Enrichment Analysis (GSEA) showing enrichment of the transcriptional signatures for osteogenesis (C and D) and proliferation (E and F) in vehicle- and siCtrl-treated cells (Ctrl + vehicle) compared to FGF2- and siCtrl-treated cells (Ctrl + FGF2) (C and E), as well as Ctrl+FGF2 cells compared to FGF2- and siY/T-treated cells (Y/T dKD + FGF2) (D and F). (**G**) Venn diagram showing the overlap of Yap-bound genes (409 genes) identified from CUT&Tag-seq with DEGs between the Ctrl + vehicle and Ctrl + FGF2 cells, as well as the DEGs between the Ctrl + FGF2 and Y/T dKD + FGF2 cells identified in the RNA-seq data from d5 osteogenic induced O9-1 NC cells. (**H**-**I**) GO analysis of candidate Yap target genes that are upregulated (H) or downregulated (I) by FGF2 and restored following Yap/Taz double knockdown (dKD).


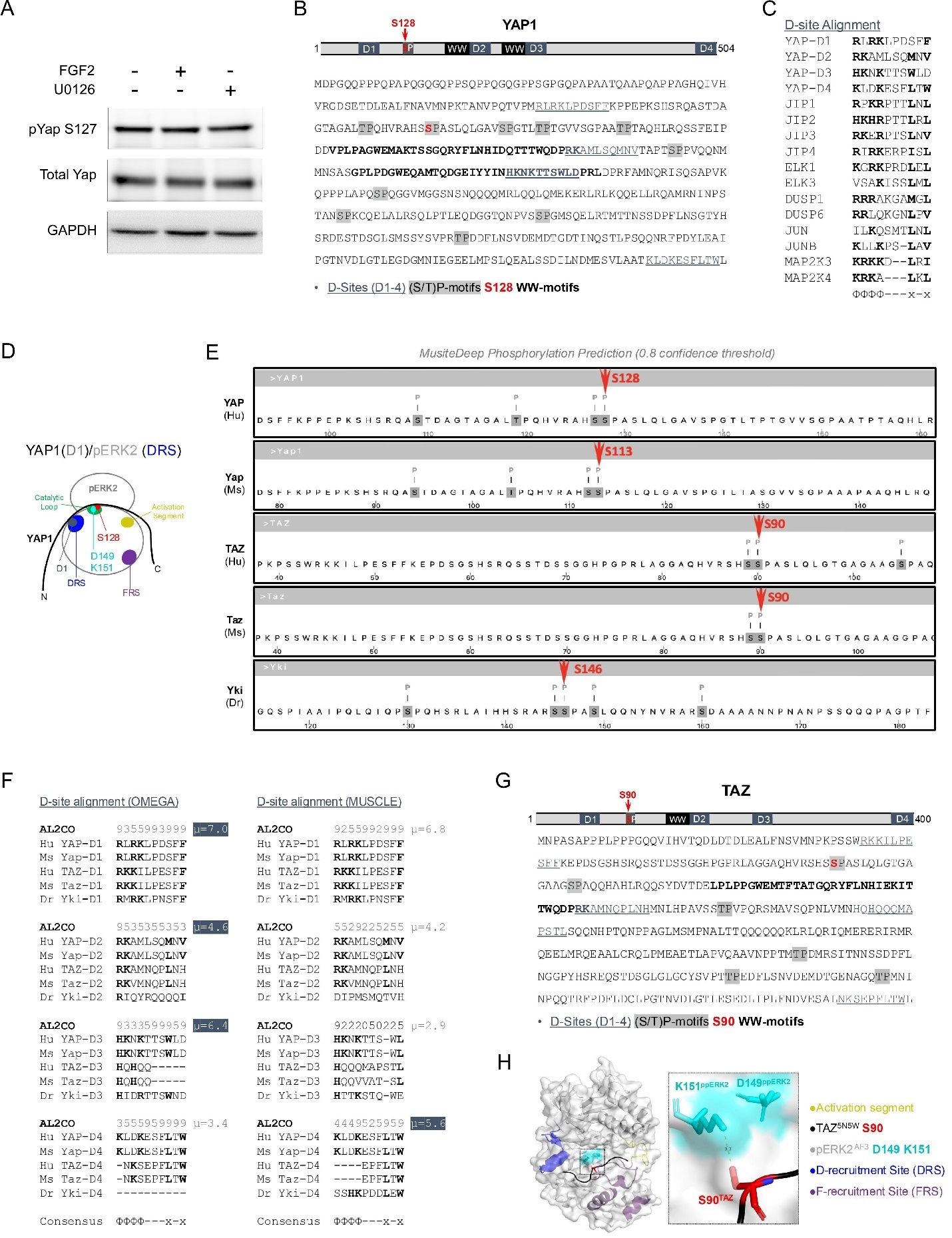


**Figure S5. Bioinformatic Prediction of YAP1-ERK2 Binding**. (**A**) Western blot for pYap S127 and total Yap expression in O9-1 NC cells following FGF2 or pERK1/2 inhibitor, U0126, treatment. GAPDH is used as a loading control. n = 3 independent experiments. (**B**) Amino acid sequence and domain organization of human YAP (UniProt: P46937), showing ten candidate (S/T)P motifs (grey), WW motifs (bold black; residues 171-204 for WW1 and 230-263 for WW2), four D-sites (navy blue, underscored), and the S128 residue (red) known to be phosphorylated by NLK, a kinase at the same level as ERK2. (**C**) Alignment of YAP’s four candidate D-sites with known MAPK substrates (adapted from Futran et al., 2013 and Whisenant et al., 2015). D-site positions: D1 - residues 87-96 (RLRKLPDSFF); D2 - residues 203-212 (RKAMLSQMNV); D3 - residues 251-260 (HKNKTTSWLD); D4 - residues 494-503 (KLDKESFLTW). (**D**) Schematic showing the potential interface of YAP-S128 (red) with ppERK2’s D149 and K151 (cyan) stabilized by YAP’s D1 site binding to ppERK2 DRS. (**E**) Phosphorylation sites (grey colored and labeled “P”) predicted by MusiteDeep (confidence threshold set to a stringent 0.80) are shown for human (Hu), mouse (Ms), and Drosophila FGF(Dr) YAP/TAZ proteins. UniProt sequences used as input for MusiteDeep: YAP(Hu)-P46937, Yap(Ms)-P46938, TAZ(Hu)-Q9GZV5, Taz(Ms)- Q9EPK5, Yki(Dr)- Q45VV3. Red arrows indicate orthologous serine positions: YAP(Hu)-S128, Yap(Ms)-S113, TAZ(Hu)-S90, Taz(Ms)-S90, and Yki(Dr)-S146. (**F**) Extended version of YAP’s four candidate D-sites across species aligned with known MAPK substrates (adapted from Futran et al., 2013 and Whisenant et al., 2015). Alignments of YAP(Hu), Yap(Ms), TAZ(Hu), Taz(Ms), and Yki(Dr) show that D1-D3 can be resolved using standard Clustal Omega, whereas D4 can only be resolved using the more stringent MUSCLE tool. Nonetheless, the groups with the highest AL2CO mean positional conservation index (μ) and similar alignment motifs were used in downstream analyses (e.g. D1/D2/D3 from Clustal Omega and D1/D2/D4 from MUSCLE). (**G**) Amino acid sequence and domain organization of TAZ(Hu) (UniProt: Q9GZV5) showing six candidate (S/T)P motifs (grey), one WW motif (bold black; residues 124-157), four D-sites (navy blue, underscored), and the S90 residue (red) which is orthologous to YAP-S128. D-site positions: D1 - residues 44-53 (RKKILPESFF), D2 – residues 156-165 (RKAMNQPLNH), D3 (non-conserved) – residues 93-97 (HQHQQ-----), and D4 – residues 391-399 (NKSEPFLTW). (**H**) ZDOCK prediction of TAZ-S90 with ppERK2. Domains shown on ERK2 adapted from Lee et al. 2011: DRS (blue) residues – 108 to 129 and 158 to 162; FRS (purple) residues – 183 to 206, 233 to 243, and 258-265; catalytic loop residues – 148 to 154 with D149 and K151 shown (cyan); activation segment (yellow) residues – 170 to 196.


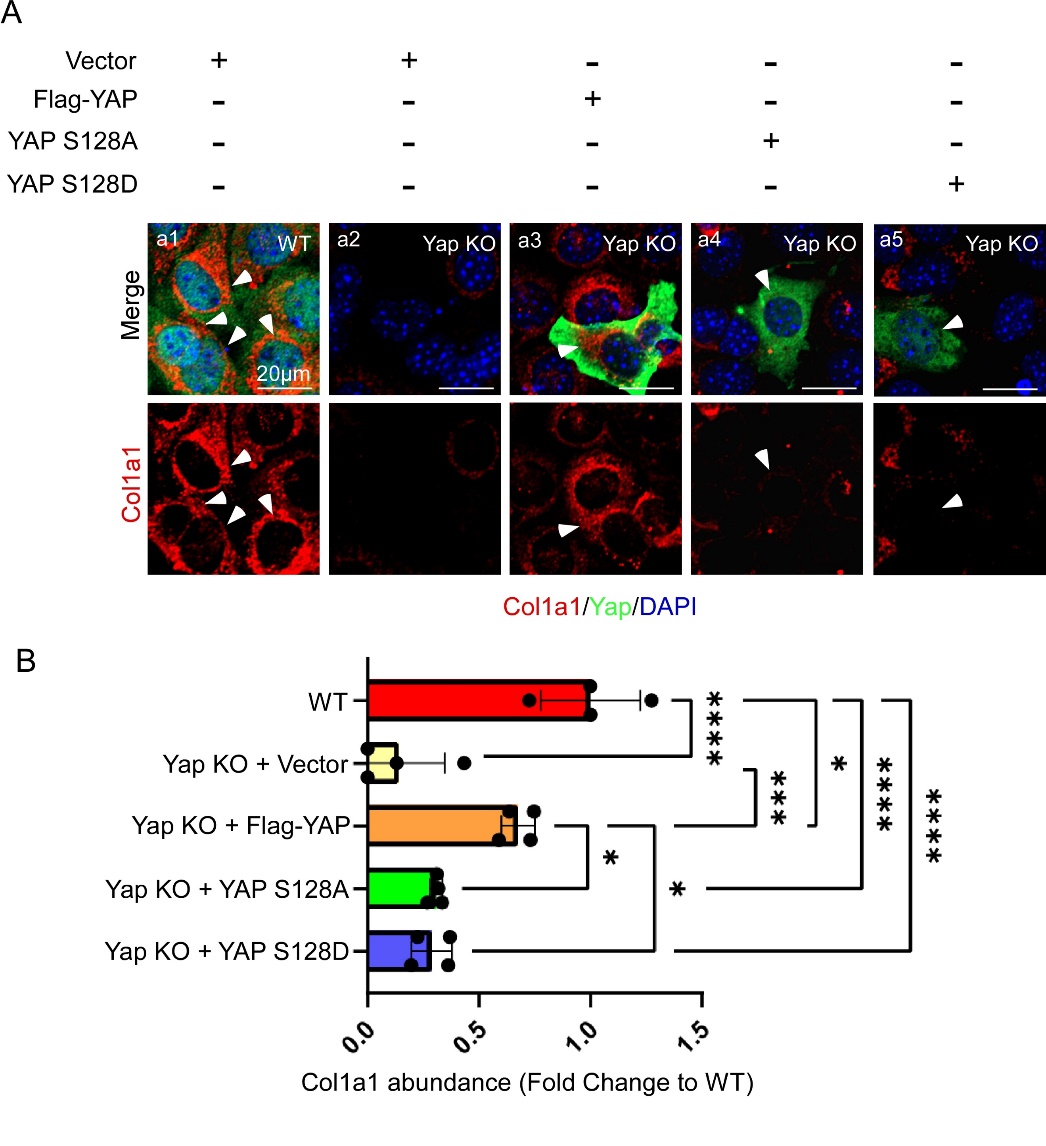


**Figure S6. Yap S128A and Yap S128D mutations impair O9-1 NC cell osteoblast differentiation.** Col1a1 (red) and Yap (green) immunofluorescence staining (**A**) of WT (**a1**) and *Yap* KO (**a2-5**) O9-1 NC cells transfected with an empty vector (**a1-2**), wildtype Flag-YAP (**a3**), or the YAP S128A (**a4**) or YAP S128D (**a5**) mutated plasmid on day 5 of osteoblast differentiation, with the corresponding quantification (**B**). Cells are counterstained with DAPI. n = 4 independent experiments. Scale bar, 20 μm. Data represent mean ± SEM. ANOVA combined with Tukey’s multiple comparisons test was used for B. *p < 0.05, **p < 0.01, ***p < 0.001, and ****p<0.0001


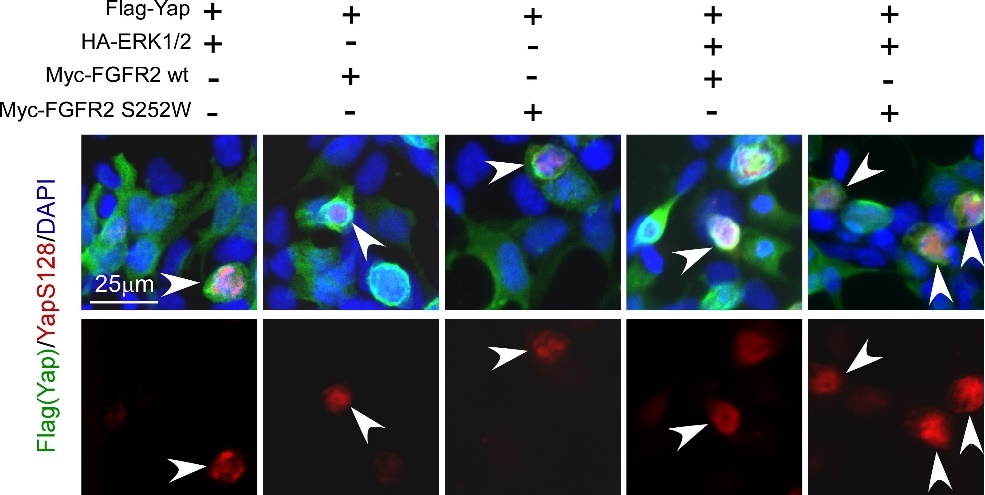


**Figure S7. FGFR2 S252W Mutation Promotes pYapS128 Expression**. Flag (Yap, green) and pYapS128 (red) co-IF staining in 293T cells transfected with the indicated plasmids. Cells are counterstained with DAPI (blue). n = 3 independent experiments. Scale bar, 25 μm.

| **Statistical Test Used** | **Figure Number** | **Software** |
| --- | --- | --- |
| Unpaired t-test with Welch’s correction | 1D, 1G, 1M, 1O, 1Q, 3B, 3D, 4E, 4G, 5F, 5H, 8F | GraphPad Prism 10.5.0 |
| One-way analysis of variance (ANOVA) with Tukey’s multiple comparison test | 2B, 2E, 2F, 2G, 2H, 3G, 3I, 3N, 4C, 5D, 8C, 8H, 8J, 8L, 8N | GraphPad Prism 10.5.0 |

**Table S1. Statistical tests used for data quantification.** Unpaired t-test with Welch’s correction was used to perform quantification between two groups, assuming the variance between the groups is different. One-way analysis of variance (ANOVA) with Tukey’s multiple comparison test was used to quantify a significant difference between three or more group means.
